# G4-DNA Formation in the *HRAS* Promoter and Rational Design of Decoy Oligonucleotides for Cancer Therapy

**DOI:** 10.1371/journal.pone.0024421

**Published:** 2011-09-08

**Authors:** Alexandro Membrino, Susanna Cogoi, Erik B. Pedersen, Luigi E. Xodo

**Affiliations:** 1 Department of Medical and Biological Science, School of Medicine, Udine, Italy; 2 Nucleic Acid Center, Institute of Physics and Chemistry, University of Southern Denmark, Odense, Denmark; Center for Genomic Regulation, Spain

## Abstract

*HRAS* is a proto-oncogene involved in the tumorigenesis of urinary bladder cancer. In the *HRAS* promoter we identified two G-rich elements, *hras*-1 and *hras*-2, that fold, respectively, into an antiparallel and a parallel quadruplex (*qhras*-1, *qhras*-2). When we introduced in sequence *hras*-1 or *hras*-2 two point mutations that block quadruplex formation, transcription increased 5-fold, but when we stabilized the G-quadruplexes by guanidinium phthalocyanines, transcription decreased to 20% of control. By ChIP we found that sequence *hras*-1 is bound only by MAZ, while *hras*-2 is bound by MAZ and Sp1: two transcription factors recognizing guanine boxes. We also discovered by EMSA that recombinant MAZ-GST binds to both *HRAS* quadruplexes, while Sp1-GST only binds to *qhras*-1. The over-expression of MAZ and Sp1 synergistically activates *HRAS* transcription, while silencing each gene by RNAi results in a strong down-regulation of transcription. All these data indicate that the *HRAS* G-quadruplexes behave as transcription repressors. Finally, we designed decoy oligonucleotides mimicking the *HRAS* quadruplexes, bearing (R)-1-O-[4-(1-Pyrenylethynyl) phenylmethyl] glycerol and LNA modifications to increase their stability and nuclease resistance (G4-decoys). The G4-decoys repressed *HRAS* transcription and caused a strong antiproliferative effect, mediated by apoptosis, in T24 bladder cancer cells where *HRAS* is mutated.

## Introduction

The ras gene family consists of three functional proto-oncogenes (*HRAS*, *NRAS* and *KRAS*) that encode for guanine-binding proteins sharing a high homology (p21^RAS^) [1]. These proteins, located on the inner cell membrane through a farnesyl group [2], are active when they are bound to GTP, and inactive when GTP is hydrolyzed to GDP [3]. Ras proteins regulate cellular responses to many extracellular stimuli, including mitogens and differentiation factors [4]. The ras genes are expressed in a tissue-specific fashion: *HRAS* is highly expressed in skin and skeletal muscles, *KRAS* in colon and thymus and *NRAS* in male germinal tissue [1]. The ras genes have similar structures and sequences with five exons, the first of which is noncoding, and conserved splicing sites. The introns, instead, have different lengths and sequences [1]. The ras proto-oncogenes are converted to oncogenes by point mutations that decrease the capacity of the encoded protein to hydrolyze GTP to GDP, with the result that p21^RAS^ remains constitutively active. Hyperactivated ras proteins stimulate phosphorylation cascades including the Raf/MEK/ERK pathway which leads to uncontrolled cell proliferation [5], [6]. Mutations in the ras genes are frequently found in many human tumors [7], [8]. *HRAS* mutations are less common, but they have a high prevalence in skin papillomas and urinary bladder tumors [9]. As 80% of bladder tumors harbor *HRAS* mutations [10] and more than half of bladder tumors overexpress *HRAS* [11], both mutation and overexpression are important factors in the tumorigenesis of bladder cancer [12]. Actually, it has been recently shown that low-level expressions of constitutively active *HRAS* induced simple urothelial hyperplasia, while the doubling of the activated *HRAS* oncogene triggered rapidly growing and penetrating tumors throughout the urinary tract. Given the crucial role of *HRAS* overexpression and mutations in the tumorigenesis of bladder cancer, one attractive therapeutic strategy could be to inhibit *HRAS* transcription with molecules that are able to impair the activity of the gene promoter. For this aim we asked how *HRAS* transcription is regulated. We observed that the promoter of *HRAS* contains numerous copies of the GGGCGGG element or its complement. This G-box has been shown to interact with the Sp1 transcription factor [13], [14]. Upstream of the transcription start site (TSS) there are runs of guanines spanning over three Sp1 sites, which are potential sites for G-quadruplex formation. We thus hypothesized that the G-rich elements might play a role in transcription regulation. G4-DNA are unusual structures stabilized by planar arrays of four guanines (G-quartet) linked one to the other by Hoogsteen hydrogen bonds [15]. The edges of the terminal G-quartets are connected by loops that can vary both in length and topology, giving rise to a variety of different conformations [16]. Genome-wide analyses have revealed that runs of guanines are abundant in gene promoter regions surrounding TSS [17]–[20]. It has been theorized therefore that G4-DNA might be involved in transcription regulation [21]–[25]. Our study provides compelling evidence that *HRAS* transcription is regulated by the interplay between Sp1, MAZ and G4-DNA, which acts as a transcription repressor. On the basis of this discovery we have designed G-rich oligonucleotides specific for *HRAS* which have a strong antiproliferative effect in urinary cancer cells bearing a mutant *HRAS*. Although the cytotoxicity of certain G-rich oligonucleotides has been previously reported, their mechanism of action is not yet fully understood [26], [27]. Our study shows that the designed quadruplex-forming oligonucleotides may act by sequestering MAZ and thus impairing *HRAS* transcription. For their potent antiproliferative effect in T24 urinary bladder cancer cells, G4-decoys seem to be very promising effector drugs for urinary bladder cancer therapy.

## Results

### The *HRAS* promoter is structurally polymorphic

**Figure 1:**
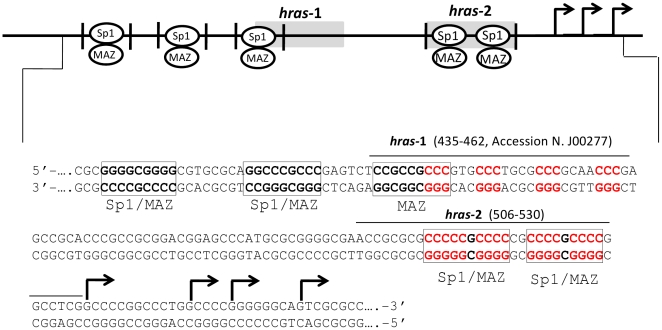
Sequence of the human *HRAS* promoter upstream of TSS. Two G-rich elements (*hras*-1 and *hras*-2) located upstream of transcription start site can potentially fold into G-quadruplex structures. The quadruplex-forming G-rich elements contain the binding sites for the transcription factors MAZ and Sp1.

The promoter of the human *HRAS* gene lacks typical TATA and CAAT boxes, contains a high G+C content (80%) and multiple copies of GGGCGGG (G-box), recognized by the transcription factor Sp1 [13], [14]. The three G-boxes closest to the RNA start sites overlap quadruplex-forming sequences, namely *hras*-1 (435–462, accession number J00277) and *hras*-2 (506–530, J00277) (Figure 1). According to a recent study, quadruplex-forming sequences covering Sp1 binding elements are present in several genes [28]. We have obtained a first hint that the *HRAS* promoter is structurally polymorphic while sequencing the expression vectors specially constructed for this study. When sequencing primer-extension reactions were performed with primers complementary to the G-rich strand, Taq polymerase unexpectedly arrested at the *hras*-2 or *hras*-1 G-rich elements. In contrast, with primers complementary to the C-rich strand we did not observe any impediment. This suggested that both *hras*-1 and *hras*-2 sequences formed unusual structures. Our hypothesis was confirmed by polymerase-stop assays. We designed two linear wild-type templates containing *hras*-1 or *hras*-2 and one mutant template in which four G→T mutations were introduced into *hras*-2 to prevent quadruplex formation. Primer-extension reactions showed that Taq polymerase in the presence of potassium arrested at the 3′ end of *hras*-2 or *hras*-1, just before the first run of guanines, in keeping with the formation of a G-quadruplex structure by each G-rich element (Figure S1).

### Promoter sequences *hras*-1 and *hras*-2 form stable G4-DNA structures in vitro

**Figure 2:**
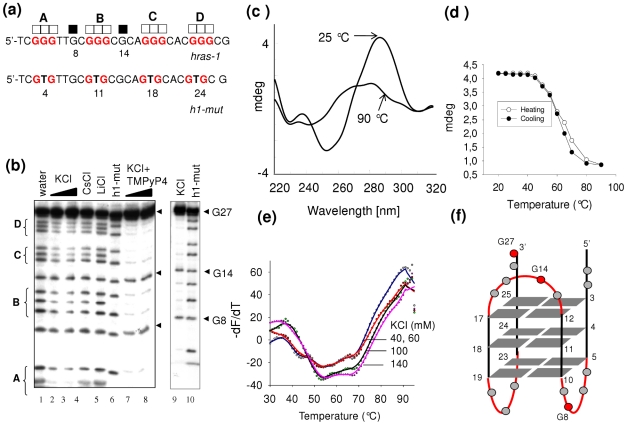
The G-rich element called *hras*-1 forms an antiparallel G-quadruplex structure. (a) Wild-type (*hras*-1) and mutated (*h1-mut*) sequences analyzed by DMS-footprinting. Full squares indicate DMS-reactive guanines, open squares indicate DMS-unreactive guanines; (b) DMS-footprinting of *hras*-1 in water (lane 1); *hras*-1 in 50 and 100 mM KCl (lanes 2 and 3); *hras*-1 in 100 mM CsCl or LiCl (lanes 4 and 5); *h1-mut* in 100 mM KCl (lane 6); *hras*-1 in 50 mM TMPyP4, 1 mM KCl (lane 7); *hras*-1 in 50 mM TMPyP4, 100 mM KCl (lane 8); *hras*-1 in 140 mM KCl (lane 9); *h1-mut* in 100 mM KCl (lane 10); (c) CD spectra at 25 and 90°C show that *hras*-1 folds into an antiparallel G-quadruplex; (d) Melting curves for quadruplex *hras*-1 obtained plotting the 287-nm ellipticity against T. A *T*_M_ of about 63°C was obtained by heating and cooling curves; (e) FRET-melting curves of quadruplex *hras*-1 labelled with FAM and TAMRA show that it folds in two G-quadruplexes with *T*_M_ of 53 and 67°C (Ex. 475 nm, Em. 520 nm); (f) Possible conformations for *hras*-1, based on footprinting and CD data, are antiparallel quadruplexes with lateral and lateral/diagonal loops. The quadruplex with lateral loops is shown in the figure.

**Figure 3:**
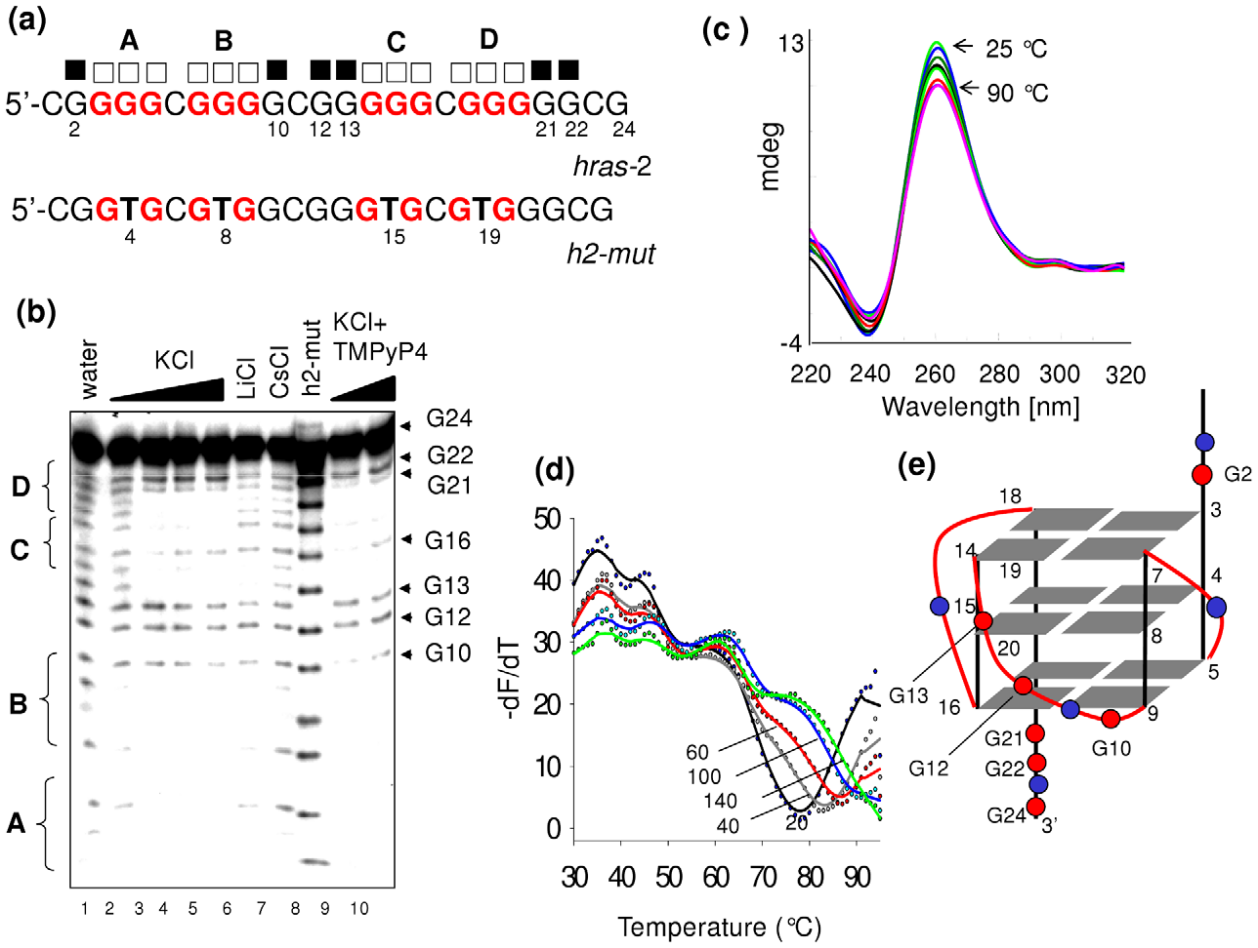
Sequence *hras*-2 forms a parallel G-quadruplex structure. (a) wild-type and mutated *hras*-2 sequences analyzed by DMS-footprinting. Full squares indicate DMS-reactive guanines, open squares indicate DMS-unreactive guanines; (b) DMS-footprinting of *hras*-2 in water (lane 1); *hras*-2 in 1, 10, 50, 100 mM KCl (lanes 2–5); *hras*-2 in 100 mM LiCl or CsCl (lanes 6,7); *h2-mut* in 100 mM KCl (lane 8); *hras*-2 in 50 nM TMPyP4, 1 mM KCl (lane 9); *hras*-2 in 50 nM TMPyP4, 100 mM KCl (lane 10). Guanines G10, G12, G13 and G21, G22 are cleaved while the other guanines are not (except G16 showing a low reactivity). Mutant *h2-mut* does not show any footprinting in 100 mM KCl; (c) CD spectra of quadruplex *hras*-2 at various temperatures between 25 and 90°C suggest the formation of an antiparallel G-quadruplex; (d) FRET-melting of quadruplex *hras*-2 at 20, 40, 60, 100 and 140 mM KCl shows only one transition with T_M_ of 78, 82, 85, >90, >92°C; (e) Possible G-quadruplex structure for *hras*-2, a parallel conformation with 1/1/4 or 1/2/3 loops, based on footprinting and CD data.

An insight into the G-quadruplexes formed by the *HRAS* G-rich elements was obtained by DMS-footprinting, circular dichroism (CD) and fluorescence resonance energy transfer (FRET) experiments. Figure 2 shows the results obtained with sequence *hras*-1. The DMS-footprints of 27-mer *hras*-1 in water or buffer containing 100 mM Li^+^ or Cs^+^ (lanes 1, 4, 5) show that all guanines react with DMS. Instead, in the presence of 50, 100 or 140 mM KCl (lanes 2, 3, 9), the guanines of the G-runs A–D are progressively protected, while guanines G8 and G14 in the intervening “TTGC” and “CGCA” sequences are not. This cleavage pattern suggests that *hras*-1 folds into a G-quadruplex (*qhras*-1). In the presence of 50 nM TMPyP4, *hras*-1 gives a strong footprint either at low (1 mM) or high (100 mM) KCl concentration (lanes 7, 8) [29]. As expected, the mutant sequence *h1-mut*, bearing *hras*-1 with four G→T mutations that abolish the folding, does not give any footprint. To determine the strand orientation of *qhras*-1 we used CD (Figure 2c,d). The CD spectrum of *qhras*-1 in 100 mM KCl is characterized by positive and negative ellipticities at 287 and 260 nm, respectively, typical of an antiparallel conformation [30]. Heating the sample we obtained a melting curve (287 nm ellipticity *versus* temperature) that was not perfectly superimposable with the cooling curve, as the first was slightly biphasic while the second was monophasic. A more sensitive method of analysis was obtained by FRET-melting experiments with sequence *hras*-1 end-labelled with FAM and TAMRA at the 5 and 3′ end, respectively. We obtained a well resolved biphasic melting curve with *T*_M_'s at 53 and 67°C indicating that *hras*-1 folds in at least two quadruplexes (Figure 2e). When the concentration of *hras*-1 was increased one order of magnitude from 200 nM to 2 µM, the *T*_M_'s did not change: a behavior typical of an intramolecular structure (not shown). Together, these data suggest that *hras*-1 adopts an antiparallel quadruplex which could assume different topologies: the one with lateral loops is shown in Figure 2f [16]. Although we know by footprinting and CD data the guanines that are involved in the formation of antiparallel *qhras*-1, an insight into structure of this quadruplex will only be obtained by NMR. Figure 3 shows the results obtained with sequence *hras*-2. The DMS-footprints of 24-mer *hras*-2 shows that in 10, 50, 100 and 140 mM KCl (lanes 2–5), the G-runs A–D are protected from DMS, while G10, G12, G13, G21 and G22 react with DMS. This indicates that the guanine triads forming the quadruplex should be G3-G4-G5, G7-G8-G9, G14-G15-G16, G18-G19-G20. The fact that G16 shows some reactivity to DMS suggests that the pentaguanine G12-G16 stretch can participate to the quadruplex either with G14-G15-G16 or with G13-G14-G15. The mutant *h2-mut* sequence and wild type *hras*-2 in 100 mM Li^+^ or Cs^+^ did not give any footprint (lanes 6–8). The footprinting in the presence of 50 nM TMPyP4 is very strong (lanes 9, 10). The CD spectrum of *hras*-2 shows a strong ellipticity at 260 nm indicative of a G-quadruplex with a parallel conformation (Figure 3c) [30]. This structure is so stable that the CD at 85°C still shows an intense 260 nm ellipticity. The stability at various KCl concentrations was determined with sequence *hras*-2 labeled with FAM and TAMRA. We performed FRET-melting experiments at 20, 40, 60, 100 and 140 mM KCl and obtained *T*_M_ values of 78 to 82, 85, >90 and >92°C, respectively (Figure 3d). In this case, too, the *T*_M_'s did not change when the concentration was increased one order of magnitude (from 200 nM to 2 µM) (not shown). Overall, our data demonstrate that *hras*-2 forms a parallel G-quadruplex (*qhras*-2) whose putative structure inferred by DMS-footprinting and CD should have either 1/1/4 or 1/2/3 loops (Figure 3e). A definitive structure assignment will only be possible on the basis of NMR data.

### G4-DNA destabilizing point mutations in *hras*-1 and *hras*-2 strongly upregulate *HRAS* transcription

**Figure 4:**
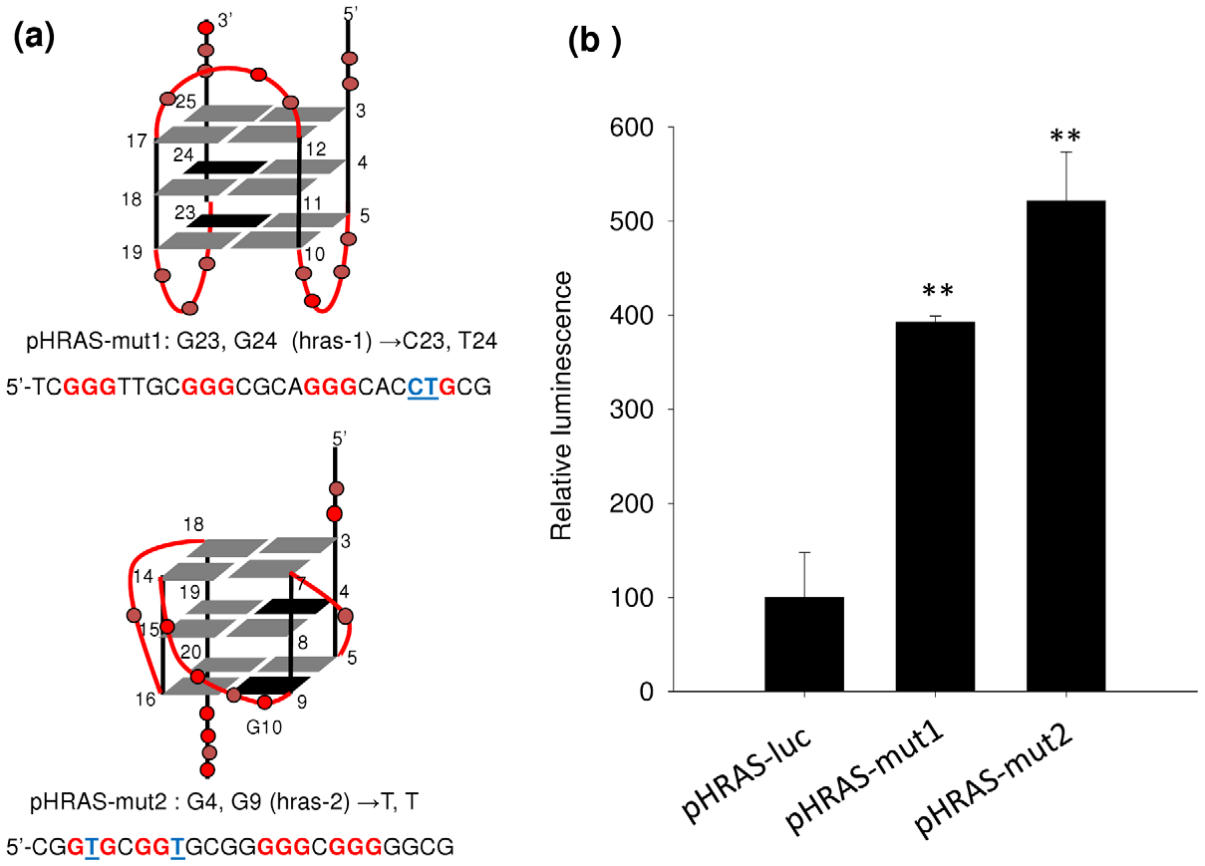
Effect of quadruplex DNA on *HRAS* transcription. (a) Two G4-DNA destabilizing point mutations in the *hras*-1 or *hras*-2 element have been introduced. Plasmid pHRAS-mut1 contains a mutated *hras*-1 element, while plasmid pHRAS-mut2 contains a mutated *hras*-2 element; (b) Dual luciferase assay showing that the point mutations cause up to a 5-fold increase in *HRAS* promoter activity (see Figure 5 legend).

As sequences *hras*-1 and *hras*-2 are located immediately upstream of the transcription start site and form *in vitro* stable G-quadruplexes, we asked what happens to *HRAS* transcription when the capacity of quadruplex formation by sequences *hras*-1 and *hras*-2 is abolished. To address this point we constructed a plasmid, pHRAS-luc, bearing firefly luciferase driven by the *HRAS* promoter. From pHRAS-luc we obtained by site-directed mutagenesis two mutant plasmids: pHRAS-mut1 and pHRAS-mut2, where two guanines in the second and third G-tetrads of the putative quadruplexes were replaced with thymine/cytosine or thymines (Figure 4a). These mutations abrogated the capacity of sequences *hras*-1 and *hras*-2 to fold into a quadruplex (Figure S2). The wild-type and mutant plasmids were co-transfected in HeLa cells with pRL-CMV, a vector encoding for *Renilla* luciferase. Forty-eight hours after transfection we measured firefly and *Renilla* luciferases activity in the lysates of untreated and treated cells. The results reported in Figure 4b show that blocking quadruplex formation causes a dramatic increase of firefly luciferase expression, up to 5-fold compared to control. This strongly indicates that the G-quadruplexes formed by sequences *hras*-1 and *hras*-2 are structural elements of the *HRAS* promoter that behave as repressors, as observed for the *CMYC* gene [21].

### G4-DNA stabilizing guanidinium phthalocyanines repress *HRAS* transcription

**Figure 5:**
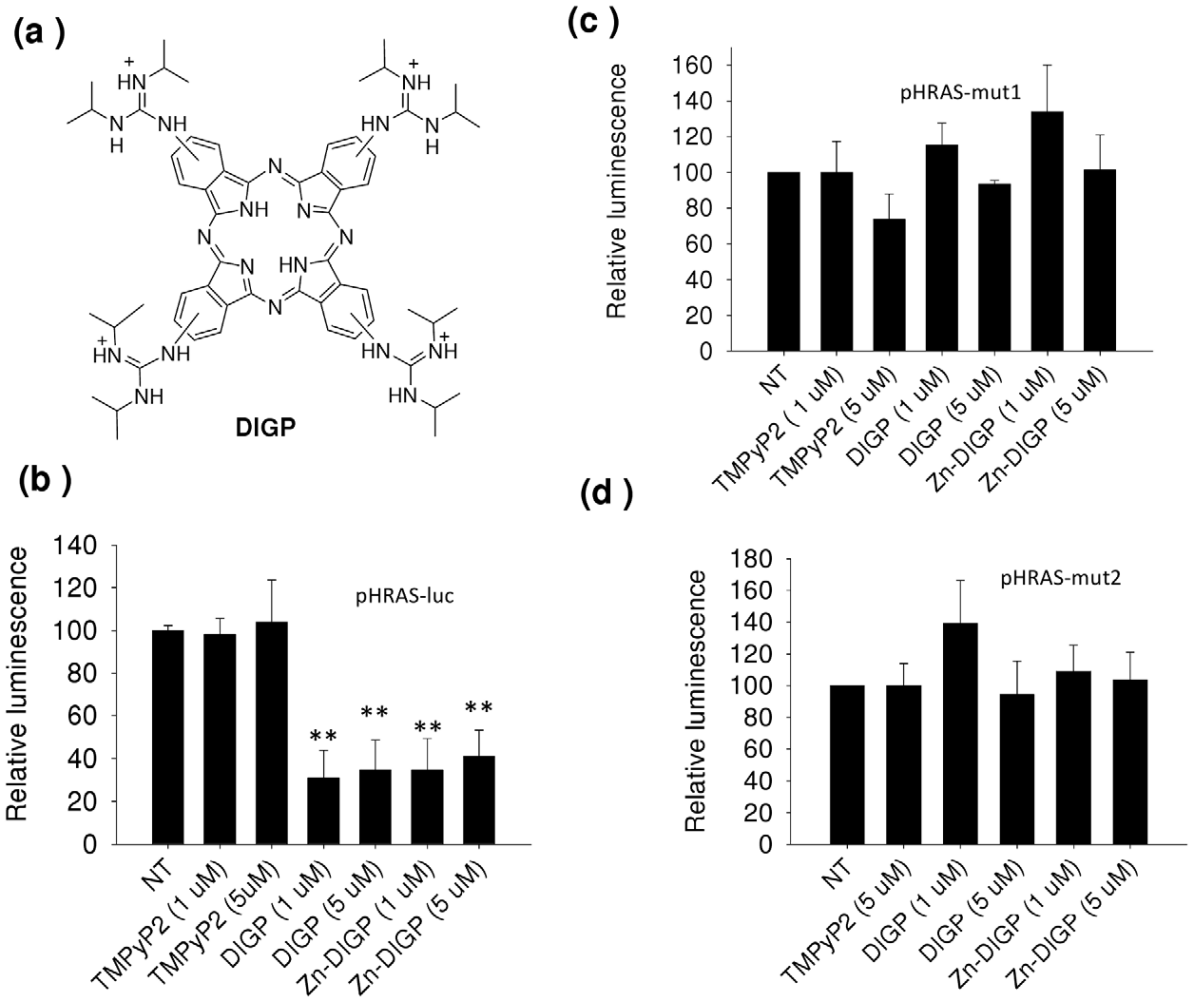
Effect of quadruplex-stabilizing ligands on *HRAS* transcription. (a) Structure of tetrakis-(diisopropyl-guanidine) phthalocyanine (DIGP) used, with its Zn-containing analogue Zn-DIGP, for luciferase experiments; (b) Dual luciferase assays showing that guanidinium phthalocyanines DIGP and Zn-DIGP, stabilizing *hras*-1 and *hras*-2 quadruplexes, repress *HRAS* promoter activity. This effect is not observed with the mutant plasmids pHRAS-mut1 and pHRAS-mut2 (control) (panels c and d). Relative luciferase is given by R_T_/R_NT_×100, where R_T_ and R_NT_ are (firefly luciferase)/(*Renilla* luciferase) in T24 cells treated with phthalocyanines and untreated cells. Differences from the control are supported by Student's *t* test, P<0.05 (one asterisk), P<0.01 (two asterisks).

Given that quadruplex DNA behaves as a repressor element for *HRAS*, we examined the effect on transcription of G4-DNA stabilizing ligands. Due to their specificity for G4-DNA, in our experiments we used as ligands modified phthalocyanines: tetrakis-(diisopropyl-guanidine) phthalocyanine (DIGP) and its Zn-containing derivative Zn-DIGP (Figure 5a) [31]–[33]. HeLa cells were first treated with 1 or 5 µM DIGP or Zn-DIGP for 24 h and then co-transfected with a mixture of pHRAS-luc and pRL-CMV. After transfection, the cells were let to grow for 48 h before firefly and *Renilla* luciferases activity were measured with a luminometer. As a control (i) we treated the cells with TMPyP2, a porphyrin that does not bind to G4-DNA [34]; (ii) we used as a reporter vector pHRAS-mut1 or pHRAS-mut2 bearing a mutated G-element unable to form a quadruplex structure. Figure 5b, c, d shows that DIGP and Zn-DIGP strongly inhibit luciferase from wild-type pHRAS-luc, while TMPyP2 does not. Moreover, the phthalocyanines do not inhibit luciferase in the cells treated with the mutant plasmids pHRAS-mut1 or pHRAS-mut2, in keeping with the fact that the G-quadruplexes cannot be formed by these vectors. These data provide further evidence that quadruplexes *qhras*-1 and *qhras*-2 act as transcription repressors.

### Transcription factors Sp1 and MAZ bind to quadruplex forming sequences (QFS) *hras*-1 and *hras*-2

**Figure 6:**
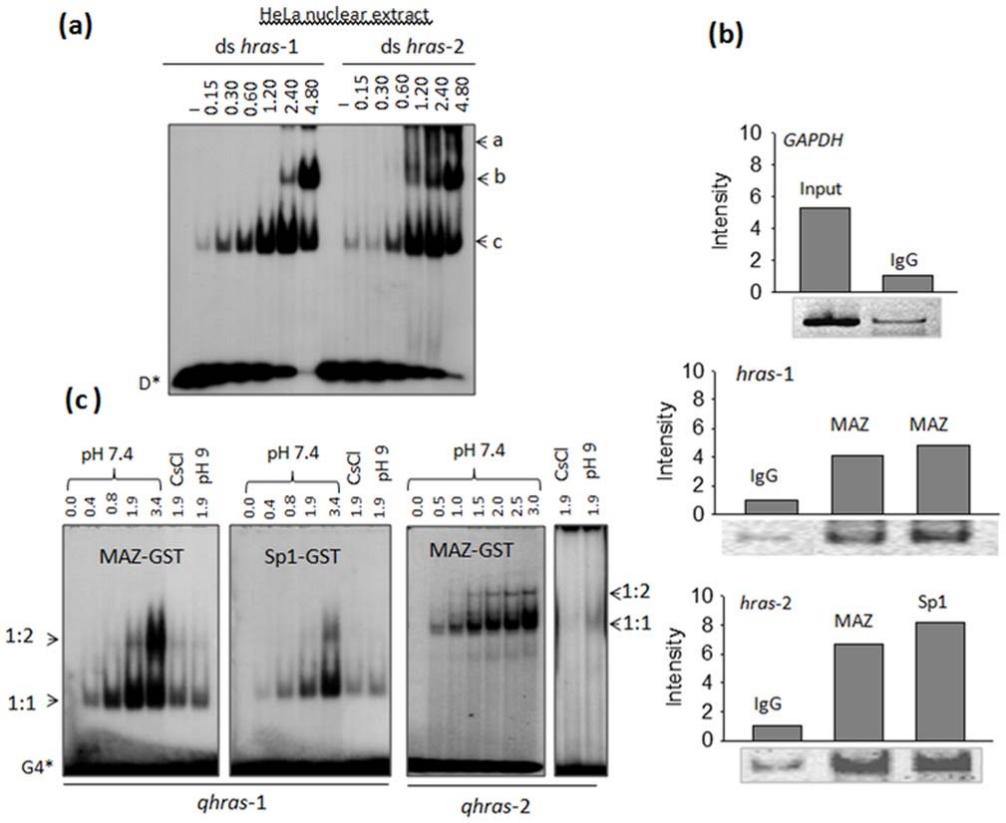
Proteins MAZ and Sp1 interact with sequences *hras*-1 and *hras*-2. (a) EMSA showing the formation of DNA-protein complexes between duplexes *hras*-1 and *hras*-2 and HeLa nuclear extract; amounts of extract used (µg) are indicated, radiolabelled DNA was about 4 nM. Letters a, b and c indicate the DNA-protein complexes; (b) Chromatin immunoprecipitation analysis showing the interaction of MAZ and Sp1 with sequences *hras*-1 and *hras*-2. The interaction between MAZ and Sp1 with the *HRAS* sequences was analyzed by a PCR amplification of 190 bp (*hras*-1) and 161 bp (*hras*-2) fragments. The two bands for complex MAZ∶hras-1 have been obtained in the presence and absence of Mg^2+^ in the mixture (c) EMSA showing the binding of MAZ-GST and Sp1-GST to quadruplexes *qhras*-1 and *qhras*-2.

As our transcription data suggest that both sequences *hras*-1 and *hras*-2 are critical for transcription, we asked if they are recognized by nuclear proteins. An answer to this question was obtained by mobility-shift assays with a HeLa nuclear extract. Figure 6a shows that both *HRAS* duplexes form with a HeLa nuclear extract distinct DNA-protein complexes, thus pointing out the relevance of these sequences for *HRAS* transcription. Ishii *et al.* showed that sequence *hras*-2, which contains two copies of GGGCGGG, is bound by Sp1 [13], [14]. However, sequences *hras*-1 and *hras*-2 should also interact with the myc associated zinc-finger transcription factor (MAZ), its binding site being (G/C)GG(C/A)GGG [35], [36]. In fact, it has been reported that MAZ and Sp1 often regulate transcription in a cooperative way [37], [38].

To prove that Sp1 and MAZ bind to sequences *hras*-1 and *hras*-2 under *in vivo* conditions, we performed chromatin immunoprecipitation (ChIP) experiments. Living HeLa cells were treated with formaldehyde to crosslink the DNA-protein complexes, chromatin was sheared into fragments and then immunoprecipitated with anti-MAZ and anti-Sp1 antibodies (Abs). The abundance of *HRAS* promoter sequences in the immunoprecipitates was measured by PCR using primers specific for sequences *hras*-1 and *hras*-2. The results reported in Figure 6b show that: IgG Ab did not immunoprecipitate DNA-protein complexes containing sequence *hras*-1 or *hras*-2 (negative control); anti-MAZ Ab did immunoprecipitate a DNA-protein complex containing *hras*-1 and *hras*-2; anti-Sp1 Ab pulled down a complex with *hras*-2. By measuring the intensity of the bands, we found that the *HRAS* sequences were more abundant in the immunoprecipitates with anti-MAZ and anti-Sp1 Abs than in the IgG Ab immunoprecipitate. We thus concluded that under *in vivo* conditions MAZ is associated to sequences *hras*-1 and *hras*-2, while Sp1 is associated to sequence *hras*-2. This was confirmed by EMSA with recombinant MAZ and Sp1 (Figure S3). As previous studies have shown that MAZ binds to G4-DNA from the murine *KRAS* [24] and *c-myb* [39] promoters, we explored whether it also binds to the *HRAS* quadruplexes. We performed EMSA with recombinant MAZ and Sp1, which were expressed in *E. coli* as GST fusion proteins (Figure S4). Figure 6c shows that at pH 7.4, 50 mM KCl, *qhras*-1 and *qhras*-2 with increasing amounts of MAZ-GST form - in the presence of 100-fold excess nonradiolabelled poly d(I-C) - two retarded bands due to the formation of two DNA-protein complexes, most likely with 1∶1 and 1∶2 stoichiometry. It is important to note that in 50 mM CsCl, where the *HRAS* sequences are unstructured (see DMS footprinting), both complexes are destabilized indicating that the DNA-protein interaction is mediated by the quadruplex structure. In a buffer at pH 9 where MAZ probably modifies its own folding, the DNA-protein interaction is also inhibited. We also tested the binding of Sp1-GST to the *HRAS* quadruplexes and found that Sp1-GST interacts with the antiparallel *qhras*-1 quadruplex, but in a weaker way compared to MAZ.

### MAZ and Sp1 synergistically activate *HRAS* transcription

**Figure 7:**
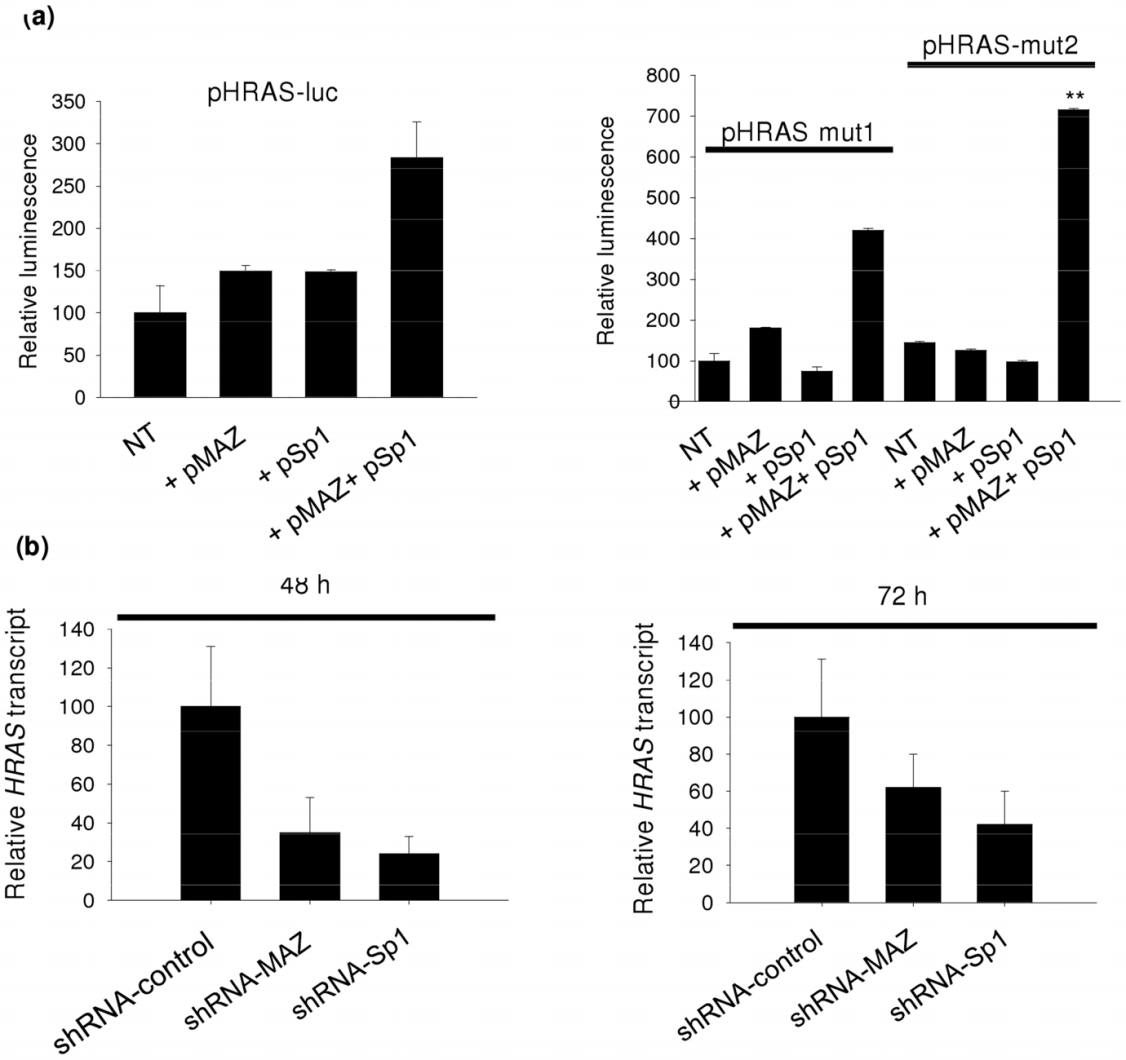
MAZ and Sp1 sinergistically activate *HRAS* promoter. (a) HeLa cells transfected with pHRAS-luc/pRL-CMV, pHRAS-luc/pRL-CMV/pMAZ; pHRAS-luc/pRL-CMV/pSp1; pHRAS-luc/pRL-CMV/pMAZ/pSp1. The dual luciferase assays were performed 24 h following transfection. Relative luminiscence is given by R_T_/R_NT_×100, where R_NT_ is (firefly luciferase)/(*Renilla* luciferase) in T24 cells treated with only pHRAS-luc and pRL-CMV, while R_T_ is (firefly luciferase)/(*Renilla* luciferase) in T24 cells treated with pHRAS-luc +pMAZ and/or pSp1+pRL-CMV. Differences from the control are supported by Student's *t* test, P<0.05 (one asterisk), P<0.01 (two asterisks); (b) Level of *HRAS* transcript in HeLa cells in which MAZ or Sp1 was knocked down by validated shRNAs.

To prove that MAZ and Sp1 are involved in *HRAS* transcription, we co-transfected plasmid pHRAS-luc in HeLa cells either with pMAZ (encoding for MAZ) or pSp1 (encoding for Sp1) or with a mixture of both plasmids (Figure 7a). When pMAZ or pSp1 was cotransfected with the reporter vector, the level of luciferase expression increased by 50% compared to control. When both pMAZ and pSp1 were cotransfected with the reporter vector, transcription increased nearly 3-fold over control, indicating that both proteins synergistically activated *HRAS* promoter. The synergy was stronger (7-fold compared to control) when pMAZ and pSp1 were cotransfected with the mutant vector pHRAS-mut1 or pHRAS-mut2, bearing mutated *hras*-1 or *hras*-2 sequences which are unable to form quadruplex structures. This suggests that transcription is activated when sequences *hras*-1 and *hras*-2 are in the unfolded duplex conformation. Furthermore, as a proof that transcription requires both MAZ and Sp1, we knocked down with validated shRNA each of the two transcription factors separately (Figure S5). HeLa cells were treated with shRNA specific for MAZ or Sp1 and the levels of *HRAS* transcripts were measured by real-time PCR, at 48 and 72 h following treatment. It can be seen that *HRAS* transcription was reduced to 30 and 20% of control, 48 h following treatment with anti MAZ and anti Sp1 shRNA, respectively (Figure 7b). Taken together our data demonstrate that *HRAS* transcription is activated synergistically by MAZ and Sp1.

### MAZ destabilizes the *HRAS* G-quadruplexes

**Figure 8:**
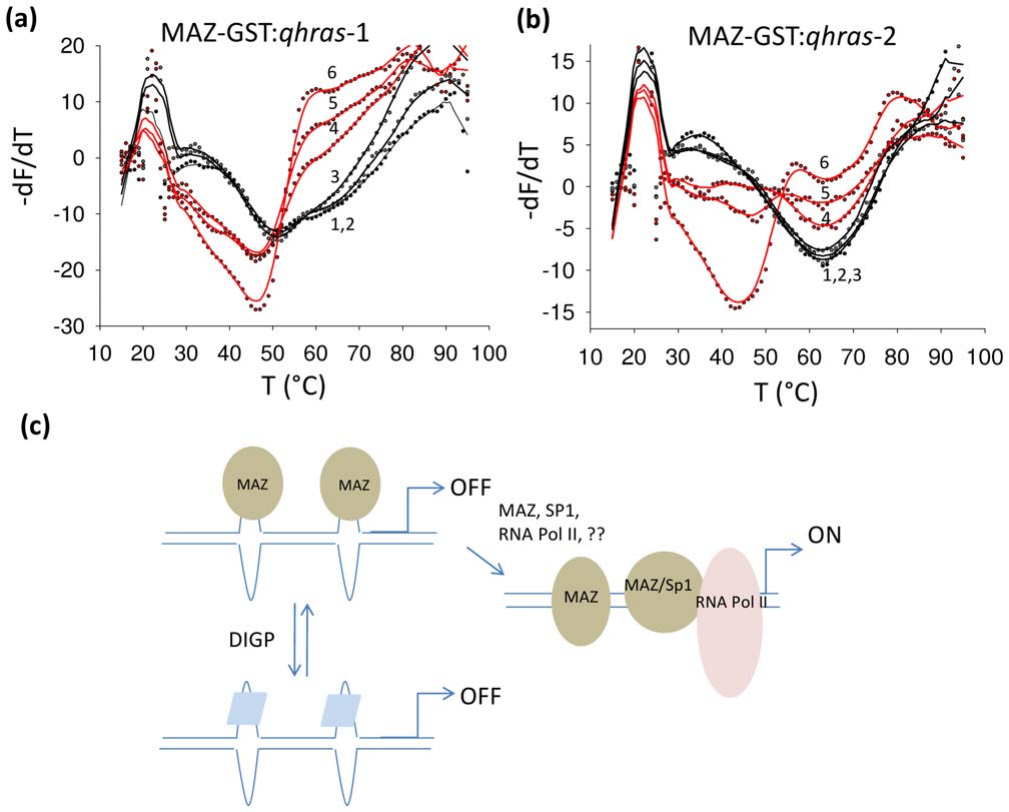
MAZ destabilizes quadruplexes *hras*-1 and *hras*-2. FRET-melting experiment showing that in 50 mM KCl, MAZ-GST at r = 2.5, 5 and 10 (r = [MAZ]/[G4-DNA]) (curves 4, 5 and 6) destabilizes quadruplex *qhras*-1 in 50 mM KCl, 50 µM Zn-acetate [curve 1 (panel a)] and quadruplex *qhras*-2 in 100 mM NaCl, 50 µM Zn-acetate [curve 1, (panel b)]. BSA and TR (trypsinogen) at r = 10 do not have any effect on *qhras*-1 and *qhras*-2 quadruplexes) (curves 2 and 3, panels a and b); (c) Schematic representation of *HRAS* transcription regulation proposed by this study. When the critical promoter elements *hras*-1 and *hras*-2 are folded, they behave as transcription repressors. This is suggested by the fact that quadruplex-destabilizing point mutations in *hras*-1 and *hras*-2 result in a significant increase of transcription, while quadruplex-stabilizing phthalocyanines are found to repress transcription. MAZ, through its capacity to recognize the quadruplex structures, should be recruited to the promoter critical region. The MAZ-DNA interaction destabilizes the G-quadruplexes, the critical *hras*-1 and *hras*-2 elements assume a duplex conformation that favors the binding of Sp1 and other proteins of the transcription machinery. These events result in the activation of transcription.

Considering that MAZ is essential for *HRAS* transcription and recognizes *qhras*-1 and *qhras*-2, we asked whether these quadruplexes are unfolded by MAZ. To answer this question, we performed FRET-melting experiments with recombinant MAZ-GST. Quadruplex *qhras*-1 end-labelled with FAM and TAMRA was incubated in 50 mM KCl, for 30 min in the presence of increasing amounts of MAZ-GST or control protein (BSA or trypsinogen). The melting curves (−dF/dT) showed that *qhras*-1 alone melts with its typical biphasic profile with *T*_M_ at 53 and 67°C (Figure 8a curve 1). But when *qhras*-1 was incubated with MAZ-GST at (moles of protein)/(moles of DNA) ratio r = 2.5, 5, 10 (curves 4, 5 and 6), the melting profile changed significantly as the *T*_M_'s fell to ∼46°C. This indicates that both *qhras*-1 quadruplexes are destabilized by MAZ-GST. As expected, unspecific proteins such as BSA or trypsinogen at r = 10 did not affect the melting of *qhras*-1 (curves 2,3). Due to its high stability in potassium (*T*_M_ = 78°C in 10 mM KCl), *qhras*-2 was analyzed in 100 mM NaCl where the *T*_M_ was 65°C. Incubated with MAZ-GST at r = 2.5, 5, 10, the quadruplex melted at 42°C, indicating that also *qhras*-2 was destabilized by MAZ-GST, but not by BSA or trypsinogen. We also ascertained that GST had no influence on the melting of the *HRAS* quadruplexes (Figure S6b). An additional control that we performed to rule out that bacterial proteins did not contribute to quadruplex destabilization was to mix Glutathione Sepharose 4B resin with an extract obtained from non-transformed BL21 DE3 plysS bacteria. An SDS-PAGE analysis of the eluate with 10 mM reduced glutathione showed that no bacterial proteins bound non-specifically to the resin (Figure S6a).

We were surprised about the unfolding activity of MAZ, as we recently reported that MAZ stabilized the murine *KRAS* quadruplex [24]. However, it should be borne in mind that MAZ having six zinc fingers can interact with DNA in a complex way, as observed with qTBP42 and CBF-A [40], [41]. These proteins disrupt the dimeric quadruplex formed by the FMR1 d(CGG)_n_ repeat but they also stabilize the telomeric quadruplex d(TTAGGG)_n_. CBF-4 and qTBP42 have four RNP domains but only two are involved in G4-DNA binding. It has been demonstrated that different RNP combinations are responsible for either the stabilizing or the destabilizing activity.

### G4-DNA decoy ODNs mimicking *HRAS* quadruplexes inhibit transcription and cell growth

**Figure 9:**
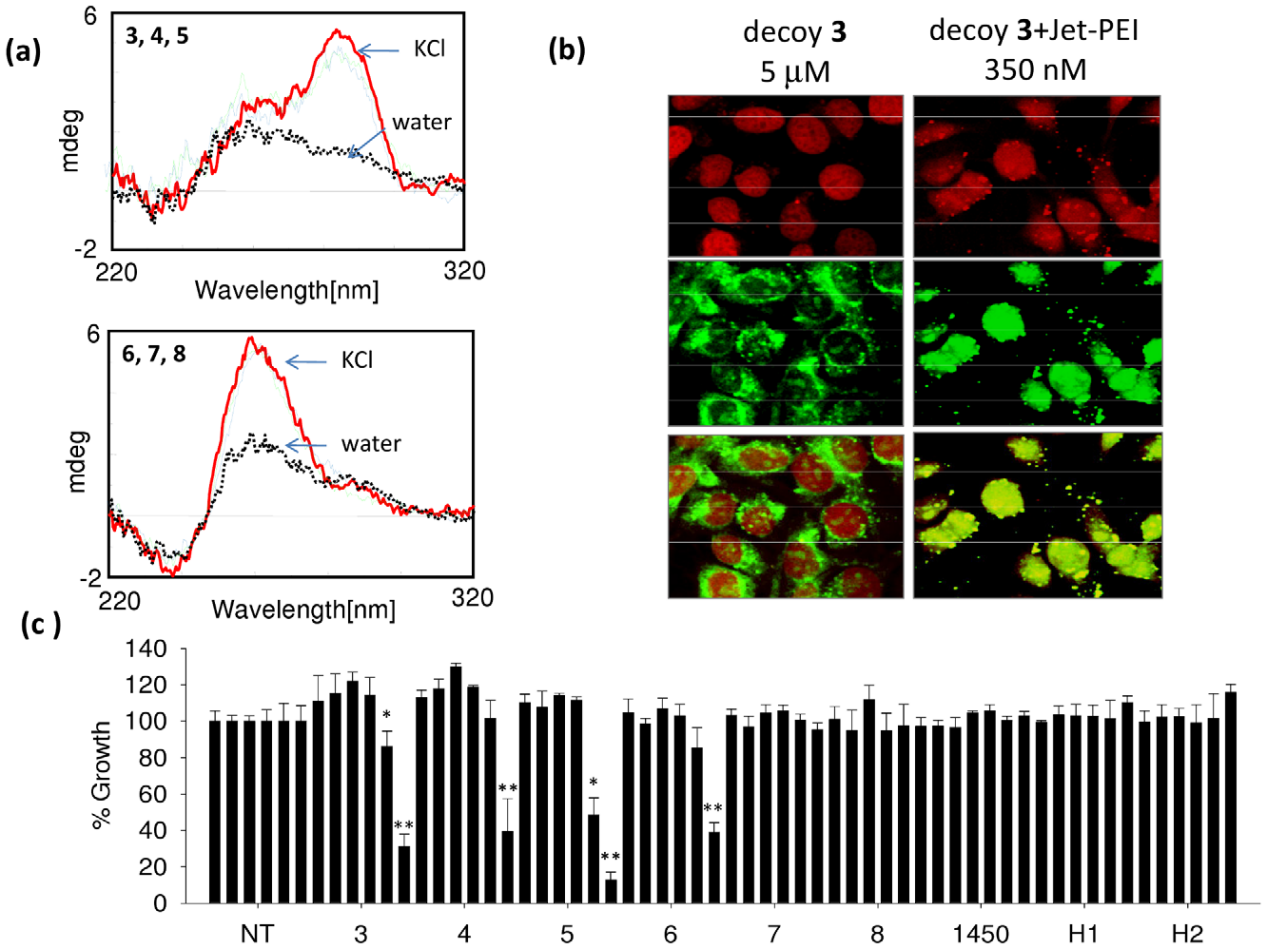
Effect on cell growth of G4-decoys. (a) CD of 2 µM G4-decoys **3**, **4**, **5**, **6**, **7**, **8** in 100 mM KCl, 50 mM Tris-HCl, pH 7.4, cuvette 0.5 cm; (b) confocal microscopy images of T24 cells treated with decoy **3** labelled with fluorescein in the presence and absence of polyethylenimmine (jet-PEI). Top panel shows the nuclei stained with propidium iodide, mid panel shows the intracellular distribution of the decoy, bottom panel shows the overlay; (c) Dose response assays in T24 urinary bladder cancer cells treated with the G4-decoys at increasing concentrations (100, 200, 300, 400, 600 and 800 nM - double delivery); differences from the control are supported by Student's *t* test, P<0.05 (one asterisk), P<0.01 (two asterisks).

Considering that *HRAS* transcription is activated by a combined action of MAZ and Sp1 and that *qhras*-1 and *qhras*-2 bind to MAZ (*qhras*-1 also binds to Sp1), we designed a decoy strategy against *HRAS* oncogene. We reasoned that these oligonucleotides mimicking quadruplexes *qhras*-1 or *qhras*-2 (G4-decoys) should sequester MAZ and inhibit *HRAS* transcription as well as cell growth (Figure 8c). To increase the stability of the G4-decoys we inserted in their sequence one or two units of (R)-1-O-[4-(1-Pyrenylethynyl) phenylmethyl] glycerol (P) to cap the quadruplex ends [42]. To increase their resistance against endogenous nucleases, we introduced LNA residues at the 3′ end and in one loop. We designed three G4-decoys mimicking quadruplex *qhras*-1 (**3**, **4** and **5**) and three mimicking quadruplex *qhras*-2 (**6**, **7** and **8**) (Figure S7) (Table 2). The latter show CD spectra similar to that of wild type *qhras*-2, with a strong ellipticity at 260 nm typical of a parallel quadruplex conformation, while the former show a CD slightly different from that of *qhras*-1, with two positive ellipticities at 260 and 287 nm, suggesting that they should form a mixed parallel-antiparallel quadruplex [43] (Figure 9a). The antiproliferative activity of the designed G4-decoys was tested in two types of cells: HeLa and T24 urinary bladder cancer cells that harbour a mutant *HRAS* [codon 12 GGC (Gly) is changed in GTC (Val)], expressing a hyperactivated HRAS protein [44]. In a first set of experiments we delivered the G4-decoys (5 µM) without any transfectant agent and did not observe any effect on cell proliferation. To see if this was due to a poor oligonucleotide uptake, we analyzed by confocal microscopy T24 bladder cells treated for 24 h with 5 µM decoy **3** covalently labelled with fluorescein (**3-F**). Figure 9b shows typical images: the nuclei stained with propidium iodide (red fluorescence), the intracellular distribution of the oligonucleotide (green fluorescence) and the overlay of both emissions. It appears clear that **3-F** localizes basically in the cytoplasm, and this is the reason why the decoys are not active when they are delivered as free molecules. We then treated the cells with **3-F** (350 nM) mixed to polyethylenimine (PEI). Figure 9b shows that despite the low concentration, **3-F** was efficiently taken up by the cells and uniformly distributed in the nucleus. We therefore decided to perform our proliferation experiments using PEI as a transfectant agent. We did a dose-response experiment by delivering the G4-decoys in two doses to T24 cells, one 48 h after the other. Three days after the first delivery, we performed a resazurin proliferation assay. It can be seen that the three G4-decoys specific for *hras*-1 (**3**, **4** and **5**) promoted a dramatic inhibition of cell growth with IC_50_ of about 700 nM (Figure 9c). In contrast, only compound **6** specific for *qhras*-2 showed some antiproliferative effect in T24 cells. Remarkably, the oligonucleotides with the sequence of *hras*-1 (H1) or *hras*-2 (H2) and control oligonucleotide 1450 (not folding into a quadruplex) did not show any antiproliferative effect (H1 and H2 are probably degraded by nucleases). A proliferation assay performed as a function of time showed that the growth inhibition promoted by the G4-decoys did not weaken over a period of 144 h (Figure S8).

**Figure 10:**
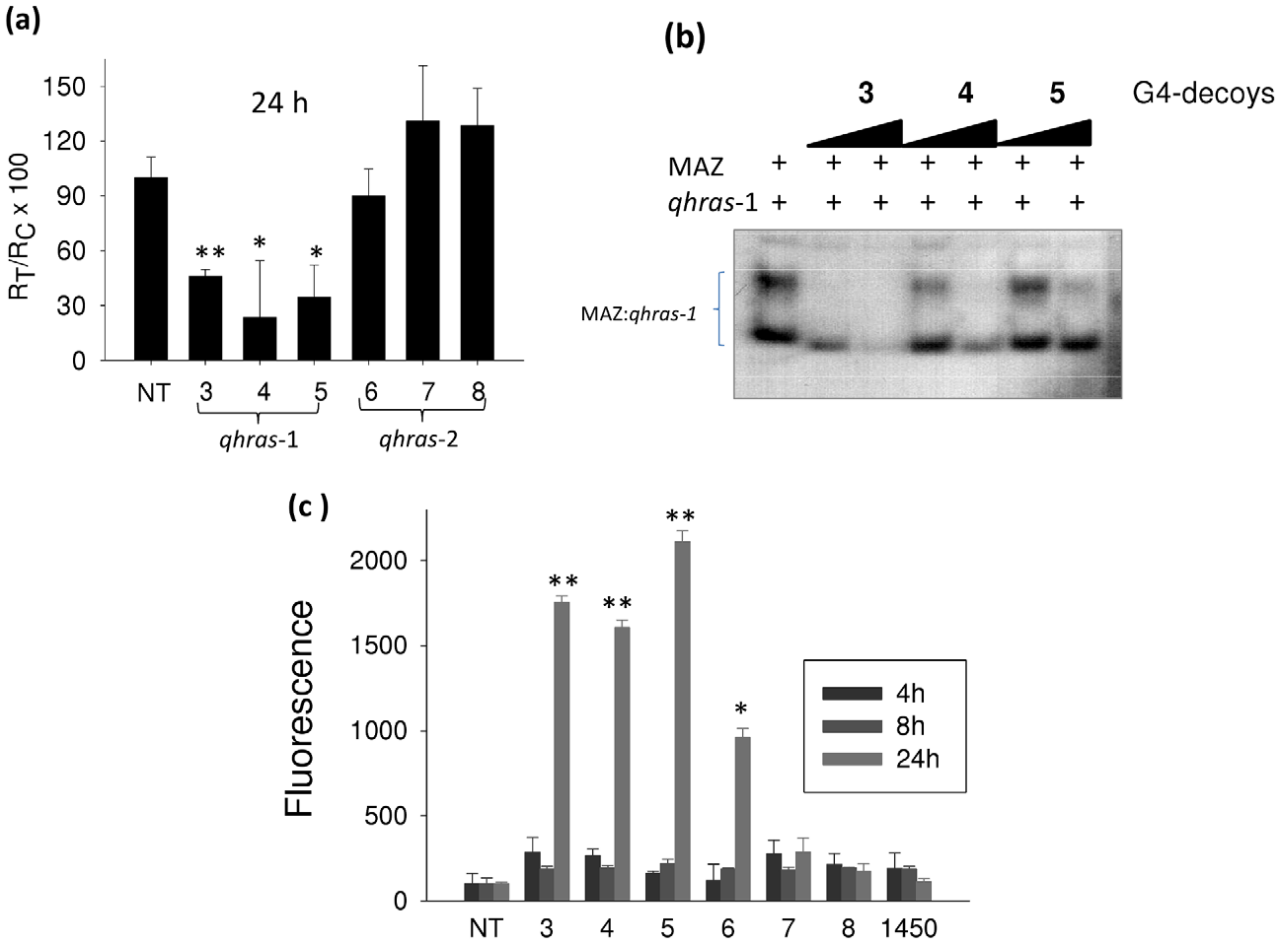
Effect of G4-decoys on *HRAS* transcription. (a) Real-time PCR determination of the *HRAS* transcripts in T24 urinary bladder cancer cells treated with 800 nM G4-decoys specific for *hras*-1 and *hras*-2 for 24 h; (b) EMSA showing that the DNA-protein complexes between 3 µg MAZ and radiolabelled *qhras*-1 (10 nM) are competed away by cold G4-decoys 10- and 50-fold in excess compared to radiolabelled *qhras*-1 quadruplex; (c) Caspase 3/7 assays showing that in T24 cells active G4-decoys **3**, **4**, **5** and **6** strongly activate apoptosis. Differences from the control are supported by Student's *t* test, P<0.05 (one asterisk), P<0.01 (two asterisks).

According to the postulated mechanism of action, the G4-decoys **3**, **4** and **5** should repress *HRAS* transcription by taking MAZ (and Sp1) away from the promoter. Actually, we found that the level of *HRAS* transcript, determined by real-time PCR 24 h after decoy treatment, was reduced up to 30% of control, in T24 cells treated with the active decoys **3**, **4** and **5** (specific for *qhras*-1). In keeping with proliferation data, the decoys **7** and **8** that are not active (specific for *qhras*-2) do not repress *HRAS* transcription (Figure 10a). This result correlates with the finding that decoys **3**, **4** and **5** strongly compete with the binding of MAZ to the *hras-1* quadruplex, suggesting that these active decoys bind to MAZ (Figure 10b).

Finally, an insight into the killing mechanism caused by the designed G4-decoys was obtained by measuring the activity of caspases 3 and 7 in untreated and decoy-treated T24 cells. Figure 10c shows that decoys with the strongest antiproliferative activity (**3**, **4** and **5**) considerably activate caspases 3/7 (24 h after treatment), suggesting that they promote cell death mediated by apoptosis.

## Discussion

The data of this study show that *HRAS* transcription is activated by Sp1 and MAZ and repressed when the binding sites of these proteins closest to TSS assume a folded G-quadruplex structure. This is a new and compelling piece of evidence pointing to a transcription mechanism which involves a simple on-off switch in a gene regulatory region where unusual G-quadruplexes behave as repressors. This was first proposed by Hurley and co-workers for the *CMYC* gene [21]. Following this transcription model we designed several decoy oligonucleotides in quadruplex conformation eliciting a potent antiproliferative effect in T24 urinary bladder cancer cells bearing mutant *HRAS*.

ChIP assays showed that the G-rich elements called *hras*-1 and *hras*-2, located upstream of TSS, are bound by the zinc-finger proteins MAZ and Sp1. MAZ interacts with these promoter sequences in a complex way, as it recognizes both duplex and quadruplex conformations of *hras*-1 and *hras*-2. The binding to the quadruplexes is catalytic in nature as *qhras*-1 and *qhras*-2 bound to MAZ go through a destabilization process that decreases the stability of the DNA-protein complexes (see EMSA). This means that PAGE gives here only an apparent affinity between MAZ and the *HRAS* quadruplexes, for which we estimated a *K*_D_ of about 1.5 µM. One should also consider that by using recombinant bacterially expressed proteins the binding data might be underestimated, as recombinant proteins do not undergo the post-translation modifications which occur in eukaryotic cells necessary for optimal binding. For instance, MAZ shows optimal binding to DNA when it is phosphorilated [45]. As a comparison, it has been estimated by a filter binding assay that recombinant nucleolin binds to parallel quadruplexes with a *K*_D_ between 79 to 367 nM and it binds to mixed parallel antiparallel quadruplexes with a *K*_D_ of 0.45–2.5 µM [46]. Instead, by surface plasmon resonance assay it has been found that recombinant nucleophosmin binds to the *CMYC* quadruplex with a *K*_D_ of 1.9 µM [47].

MAZ was identified as a G-box binding transcription factor for *CMYC* [48]. Previous studies have shown that MAZ can either activate [35]–[37], [49]–[51] or repress [39], [52] transcription. Furthermore, in certain genes MAZ regulates transcription together with Sp1, as the two proteins can bind to the same guanine blocks: the consensus sequence for MAZ is G(g/c)GGc/a GGGG(c/a)(g/t) while that of Sp1 is (g/t)GGGCGG(g/a)(g/a)(c/t) [35]. When MAZ or Sp1 was separately knocked-down with specific shRNAs, *HRAS* transcription dropped respectively to 30% or 20% of control. Conversely, when MAZ and Sp1 were over-expressed, a synergistic effect was observed and *HRAS* transcription increased 3-fold compared to control.

To explore the role of G4-DNA on *HRAS* transcription we introduced two quadruplex destabilizing G→T(C) point mutations in sequences *hras*-1 or *hras*-2 and found that transcription increased 5-fold compared to control. A similar behavior was previously reported for *CMYC* and *CMYB* genes [21], [22], [39]. Our conclusion is that both *HRAS* G-quadruplexes behave as a molecular on-off switch that either provides the binding sites to MAZ and Sp1 or subtracts them. This is also supported by the fact that guanidinium phthalocyanines stabilize the *HRAS* G-quadruplexes and repress luciferase from pHRAS-luc to 20–30% of control, but not from mutant vectors pHRAS-mut1 and pHRAS-mut2. In addition to function as a transcription factor, MAZ is also able to remove the structural blocking of transcription by the quadruplex structures, as indicated by FRET-melting data.

In the light of all these findings, we designed G4-decoys mimicking *HRAS* G-quadruplexes, which show a strong antiproliferative activity in T24 urinary bladder cancer cells harboring mutant *HRAS*. We hypothesized that the G4-decoys should take MAZ away from the promoter and inhibit *HRAS* transcription. We found that in T24 bladder cells (but also in HeLa cells, not shown) the decoys specific for quadruplex *qhras*-1 (**3**, **4**, and **5**) displayed a dramatic inhibitory activity on cell growth, at a concentration as low as 700 nM. Instead, only decoy **6** mimicking quadruplex *qhras*-2 showed some activity. In keeping with previous observations, our data suggest that quadruplex formation *per se* is not sufficient to give rise to a bioactivity, as decoys **7** and **8** though forming a stable quadruplex, are not active.

It is well known that certain G-rich oligonucleotides show a clear antiproliferative effect in cancer cells which is not due to a true antisense effect, but to their propensity to fold into a G-quadruplex [26]. How these oligonucleotides precisely work, is not yet clear, but Bates and co-workers proposed that the antiproliferative activity of certain G-rich oligonucleotides requires: nuclease resistance; efficient cellular uptake; binding to a specific protein [53]. Our G4-decoys fulfill these requirements as: their compact structure and LNA residues make them resistant to nucleases; they efficiently penetrate cell membranes and internalize in the nucleus when complexed with PEI; they interact with MAZ, an essential protein for *HRAS* transcription.

That our G4-decoys act through their binding to a nuclear protein (MAZ) is suggested by the fact that when they are delivered without a transfectant agent, they localize in the cytoplasm and are not active. In contrast, when they are delivered with PEI, they reach the nucleus and show a strong antiproliferative activity.

In accord with the proposed mechanism of action, the decoys eliciting the highest inhibition of cell growth (**3**, **4**, **5**) caused in T24 cancer cells a strong decrease of *HRAS* transcript and activation of caspases 3/7.

In summary, this work shows that: G4-DNA near the transcription start sites of *HRAS* behaves as a transcription repressor; MAZ and Sp1 bind to G-elements that can fold into quadruplex forming sequences; transcription is activated by MAZ and Sp1; MAZ destabilized the *HRAS* G-quadruplexes; G4-decoys mimicking the *HRAS* quadruplexes behave as decoy molecules against MAZ and cause a potent antiproliferative effect in T24 bladder cancer cells bearing mutant *HRAS*; the decoy strategy could provide a new therapeutic approach to treat bladder cancer.

## Materials and Methods

### Plasmids and G4-decoys synthesis

pHRAS-luc was obtained by standard cloning, while mutant plasmids pHRAS-mut1, pHRAS-mut2 were obtained by site directed mutagenesis with the gene tailor kit (Invitrogen). A 838-bp Sac I-Sac I fragment, obtained from pEJ 6.6 plasmid bearing the human *HRAS* promoter, was cut with Xma I restriction enzyme and the resulting 345 bp fragment was subcloned in pGL3–E1 basic plasmid in Sac I-Xma I upstream of firefly luciferase. In the resulting construct luciferase was driven by wild-type *HRAS* promoter (pHRAS-luc). By site directed mutagenesis we introduced in pHRAS-luc two point mutations either in *hras*-1 or *hras*-2 sequence. The primers used were 5′- CGG GGC CGA GGC CGG TGC GGT GCG TGT GCG TGT GC-3′ (for) and 5′-CCG GCC TCG GCC CCG GCC CTG GCC C-3′ (rev). PCR was performed with 3 ng/µl DNA template, 0.1 µM each primer, 0.05 units/µl AccuPrime pfx DNA polymerase (Invitrogen) in 1× AccuPrime pfx reaction mix for 3 min at 95°C, 30 cycles 1 min at 95°C, 30 s at 81°C, and 5 min at 68°C. Bacteria DIH 101 5α were transformed with PCR product, and plasmid DNA was extracted and sequenced (primer pGL3bpr 2 5′-CTT TAT GTT TTT GGC GTC TTC CA-3′).

Plasmids pCMV-MAZ (called pMAZ), pCMV-Sp1 (called pSp1), pGEX-MAZ and pGEX-Sp1 have been purchased by RIKEN (Japan).

G4-decoys were synthesized on an Expedite™ Nucleic Acid Synthesis System Model 8909 from Applied Biosystems. Purification of oligonucleotides was accomplished using a reverse-phase semipreparative HPLC on Waters Xterra™ MS C_18_ column. Oligonucleotide concentrations were determined by UV-absorbance at 260 nm, 90°C and the calculated single-stranded extinction coefficients were based on a nearest neighbour model (extinction coefficient for monomers is 22400 at 260 nm).

### CD and DMS footprinting experiments

CD spectra have been obtained with a JASCO J-600 spectropolarimeter equipped with a thermostatted cell holder, 3 µM oligonucleotides in 50 mM Tris–HCl, pH 7.4, 100 mM KCl.

DMS footprinting experiments were performed using PAGE purified oligonucleotides, 27-mer *hras*-1 and *h1-mut*, 24-mer *hras*-2 and *h2-mut* (20 nM), end-labelled with [γ-^32^P]ATP (Perkin Elmer) and polynucleotide kinase (New England Biolabs, MA). Before the reactions they were incubated overnight at 37°C, in 50 mM sodium cacodylate, pH 8, 1 µg sonicated salmon sperm DNA, 1 mM EDTA, 100 mM KCl or LiCl or CsCl or KCl+TMPyP4 as specified in figure legends. Dimethylsulphate (DMS) dissolved in ethanol (DMS∶ethanol, 4/1, vol/vol) was added to the DNA solution (1 µl to a total volume of 70 µl) and left to react for 5 min at room temperature. The reactions were stopped by the addition of 1∶4 volumes of stop solution (1.5 M sodium acetate, pH 7, 1 M β-mercaptoethanol and 1 µg/µl tRNA). DNA was precipitated with 4 volumes of ethanol and resuspended in piperidine 1 M. After cleavage at 90°C for 30 min, reactions were stopped with chilling in ice followed by precipitation with 0.3 M sodium acetate, pH 5.2 and 3 volumes of ethanol. The DNA samples were resuspended in 90% formamide and 50 mM EDTA, denatured at 90°C and run for 2 h on a denaturing 20% polyacrylamide gel, prepared in TBE and 8 M urea, pre-equilibrated at 55°C in a Sequi-Gen GT Nucleic Acids Electrophoresis Apparatus (Bio-Rad, CA), which was equipped with a thermocouple that allows a precise temperature control. After running, the gel was fixed in a solution containing 10% acetic acid and 10% methanol, dried at 80°C and exposed to Hyperfilm MP (GE Healthcare) for autoradiography.

### Cell culture and proliferation assay

HeLa and T24 urinary bladder cancer cells were maintained in exponential growth in Dulbecco's Modified Eagle's Medium (DMEM) containing 100 U/ml penicillin, 100 mg/ml streptomycin, 20 mM L-glutamine and 10% foetal bovine serum (Euroclone, Milan, Italy). For proliferative assays T24 cells were seeded (1250 cells/well) the day before decoy treatment in a 96-well plate. The G4-decoys, mixed to jetPEI (Polyplus transfection), were delivered to the cells at increasing concentrations up to 800 nM. 48 h after the first delivery a second dose of oligonucleotides was given to the cells. 72 h after the second delivery, the cell viability was measured by resazurin assays following standard procedures.

### Dual luciferase assays

Firefly luciferase activity in cell lysates was measured and normalized for *Renilla* luciferase activity using the Dual-Glo Luciferase Assay System (Promega) following vendor's instructions. Transfection was performed by mixing each vector 250 ng/well with control plasmid pRL-CMV expressing *Renilla* luciferase under control of CMV promoter 10 ng/well using Metafectene transfection reagent (Biontex Laboratories, GmbH) following manufacturer instruction. For cotransfection with pMAZ and/or pSp1 100 ng of pHRAS-luc or mutant, with 10 ng of pRL-CMV, were transfected with either 100 ng of pMAZ/pSp1 or pcDNA3 plasmid (empty vector) as mass for control transfections. Each transfection was performed in triplicate. Luciferase assays were performed 48 h after transfection following instructions. Samples were read with Turner Luminometer and expressed as Relative luminescence (see figure captions).

### Recombinant MAZ and Sp1: purification and EMSA

Recombinant MAZ and Sp1 tagged to GST were expressed in *Escherichia coli* BL21 DE_3_ plys using plasmid pGEX-MAZ and pGEX-Sp1. The bacteria were grown for 1–2 h at 37°C to an optical density at 600 nm between 0.5–2.0 prior to induction with IPTG (0.2 mM final concentration). Cells were allowed to grow overnight at 30°C before harvesting. The cells were centrifuged at 5000 rpm, 4°C, the supernatant removed and the cells washed twice with PBS. The pellet was resuspended in a solution of PBS with 1 mM PMSF, 1 µM DTT and Protease Inhibitor Cocktails (only for GST-tagged proteins) (Sigma-Aldrich). The bacteria were lysed by sonication and centrifuged for 30 min at 4°C, 16000 rpm. Glutathione Sepharose 4B (GE Healthcare) (50% slurry in PBS) was added to the supernatant from the previous step and incubated for 30 min at 4°C while shaking. The mix was centrifuged for 5 min at 500 g and the resin was washed 3 times with PBS. Proteins were eluted from the resin with a buffer composed by 50 mM Tris-HCl pH 8 and 10 mM reduced glutathione.

### Chromatin immunoprecipitation

HeLa cells were plated in 2×15 cm diameter plates, grown to 80% confluency (about 1.5×10^7^ cells) and fixed in formaldehyde 1% in PBS for 2 or 5 minutes. Chromatin immunoprecipitation assays were performed using the ChIP-IT™ Express kit (Active Motif, Rixensart, Belgium), as previously reported [24]. The antibodies used are MAZ H-50 (sc-28745, Santa Cruz, Biotechnology, Inc), Sp1 (PEP 2) (sc-59, Santa Cruz, Biotechnology, Inc) used at 20 ng/µl. The primers used are: (i) for control reaction, specific for *GAPDH* (provided by the kit) that give a 160 bp product; (ii) 5′-GGCTCCTGACAGACGGG (*hras*-1for) and 5′-GCATGGGCTCCGTCC (*hras*-1rev) giving a 190 bp product; (iii) 5′-GGACGGAGCCCATGC (*hras*-2for) and 5′-CGTATTGCTGCCGCCT (*hras*-2rev) giving a 161 bp product. Amplification products were separated by a 10% acrylamide gel in TBE and visualized with a Gel–DOC apparatus (Bio-Rad Laboratories, CA, USA).

### shRNA transfections, RNA extraction and real-time PCR

Cells were seeded 200–400000/well in a 24 well plate. shRNA plasmids were transfected the day after plating 0.5 µg/well. Plasmids used are shRNA-control, shRNA-MAZ and shRNA-Sp1 (Santa Cruz Biotechnology, CA, USA). Cells were collected 48 and 72 h after transfection.

RNA extraction: RNA was extracted using TRIzol reagent (Invitrogen, Carlsbad, CA). cDNA synthesis: 5 µl of RNA in DEPC-water was heated at 55°C and placed in ice. The solution was added with 7.5 µl of a mix containing (final concentrations) 1× buffer; 0.01 M DTT (Invitrogen); 1.6 µM primer dT [MWG Biotech, Ebersberg, Germany; d(T)_16_]; and 1.6 µM random primers (Promega); 0.4 mM dNTPs solution containing equimolar amounts of dATP, dCTP, dGTP and dTTP (Euroclone, Pavia, Italy); 0.8 U/µl RNAse OUT; 8 U/µl of M-MLV reverse transcriptase (Invitrogen). The reactions were incubated for 1 h at 37°C and stopped with heating at 95°C for 5 min. As a negative control the reverse transcription reaction was performed with a sample containing DEPC water. Real-time PCR reactions were performed with 1× iQ™ SYBR Green Supermix (Bio-Rad Laboratories, CA, USA), 300 nM of each primer, 1 µl of RT reaction. The sequences of the primers used for *HRAS*, *GAPDH*, β-2-microglobulin, Hypoxanthine Ribosyl Transferase, MAZ and Sp1 amplifications are reported in Table 1. The PCR cycle was: 3 min at 95°C, 40 cycles 10 s at 95°C, 30 s at 60°C with an iQ_5_ real-time PCR controlled by an Optical System software version 2.0 (Bio-Rad Laboratories, CA, USA).

**Table 1:** RT-PCR primers.

Oligonucleotides	5′-3′ sequence	T, °C
hHRAS for	GGG GCA GTC GCG CCT GTG AA	60
hHRAS rev	CCG GCG CCC ACC ACC ACC AG	60
hGAPDHfor	CCC TTC ATT GAC CTC AAC TAC ATG	60
hGAPDHrev	TGG GAT TTC CAT TGA TGA CAA GC	60
hHPRTfor	AGA CTT TGC TTT CCT TGG TCA GG	60
hHPRTrev	GTC TGG CTT ATA TCC AAC ACT TCG	60
hβ2µglobulinrev	CAT TCC TGA AGC TGA CAG CAT TC	60
hβ2µglobulinfor	TGC TGG ATG ACG TGA GTA AAC C	60
hmazfor	CTC CAG TCC CGC TTC T	55
hmazrev	GGG AGC AAG TCC ACC T	55
hSp1for	CCC TTG AGC TTG TCC CT	50
hSp1rev	CCT GTG AAA AGG CAC CA	50

**Table 2:** G4-decoys used for *HRAS* gene.

Oligonucleotides	5′-3′ sequence
hras-2 biotin	B-CGAGGCCGGGGCGGGGCGGGGGCGGGGGCGCGCGGT
hras-1 biotin	B-CGGCTCGGGTTGCGGGCGCAGGGCACGGGCGGC
hras-1 (H1)	TCGGGTTGCGGGCGCAGGGCACGGGCG
hras-2 (H2)	CGAGGCCGGGGCGGGGCGGGGGCGGGGGCGCGCGGT
**3**	TC**P**GGGTTGCGGGC**G**CAGGGCA CGGGC**GG**
**3-F**	F- TC**P**GGGTTGCGGGC**G**CAGGGCA CGGGC**GG**
**4**	TC**P**GGGTTGCGGGC**G**CAGGG**P**CA CGGGC**GG**
**5**	TC**P**GGGT**T**GCGGGC**G**CAGGG**P**CA CGGGC**GG**
**6**	CG**P**GGGCGGGGCGGGGGCGGGGG**CG**
**7**	CG**P**GGGCGGGGC**G**GGGGCGGGGG**CG**
**8**	CG**P**GGGCGGG**P**GCGGGGGCGGGGG**CG**
**1450**	GCGGTGTCGC**P**AAGACGCAAGACGCGGAGGCAG

B = biotin; P = TINA (S_4_); Underlined bases indicate LNA residue.

### Electrophoresis mobility-shift assays

Oligonucleotides were end-labelled with [γ-33P]ATP and T4 polynucleotide kinase. Duplexes *hras*-1 and *hras*-2 were prepared annealing (10 min at 95°C, overnight at room temperature) a mixture containing 1∶1,2 ratio of radiolabelled *hras*-1 or *hras*-2 with the complementary strand in 50 mM Tris–HCl, pH 7.4, 100 mM NaCl. Radiolabelled *hras*-1 and *hras*-2 were allowed to assume a G4-DNA structure in 50 mM Tris–HCl, pH 7.4, 100 mM KCl, by heating at 95°C and overnight incubation at 37°C. Before EMSA, the radiolabelled oligonucleotides were treated for 30 min at room temperature with different amounts of MAZ or Sp1 (or extract), in 20 mM Tris–HCl, pH 8, 30 mM KCl, 1.5 mM MgCl_2_, 1 mM DTT, 8% glycerol, 1% Phosphatase Inhibitor Cocktail I (Sigma, Milan, Italy), 5 mM NaF, 1 mM Na_3_VO_4_, 2.5 ng/ml poly [dI-dC]. After incubation, the reaction mixtures were loaded in 5% TBE (1X) polyacrylamide gel, thermostatted at 20°C. After running, the gel was dried and exposed to autoradiography (GE Healthcare, Milan) for 24–36 h at −80°C.

### FRET-melting experiments

FRET melting experiments were performed on a real-time PCR apparatus (CFX 96, BioRad, Hercules, CA), using a 96-well plate filled with 50 µl solutions of dual-labelled *qhras*-1 and *qhras*-2, in 50 mM Tris–HCl, pH 7.4, 50 mM KCl (*qhras*-1) or 100 mM NaCl (*qhras*-2). The protocol, used for the melting experiments, is the following: (i) equilibration step of 5 min at low temperature (20°C); (ii) stepwise increase of the temperature of 1°C/min for 76 cycles to reach 95°C. All the samples in the wells were melted in 76 min.

### Caspase assays

We performed Caspase activity assays using Apo-ONE™ Homogeneous Caspase-3/7 Assay (Promega), according to the manufacturer's protocol using a Microplate Spectrofluorometer System (Molecular Devices, Concorde, Canada).

## Supporting Information

Figure S1 Polymerase stop assays with a wild-type DNA template containing *hras*-1 or *hras*-2 and a mutant template in which four G→T point mutations were introduced in sequence *hras*-2 to abrogate quadruplex formation. Taq polymerase is arrested at the 3′ end of *hras*-2, before the first run of guanines, in keeping with the formation of a quadruplex structure by *hras*-2 (experimental conditions: 37°C, 140 mM KCl, 50 mM Tris-HCl pH 7.4) When the DNA template is incubated with G4-DNA ligands that stabilize quadruplex DNA, Taq polymerase is completely arrested and only the truncated product is produced. This is observed with porphyrin TMPyP_4_ and guanidine phthalocyanines DIGP and Zn-DIGP at r = 4 (r = [ligand]/[template]). Instead, TMPyP_2_, which does not bind to G4-DNA does not affect the processivity of Taq polymerase. When the experiment is performed with the mutated template, Taq polymerase does not stop at the G-element and full product is observed. A longer truncated product is observed with the mutated template, probably due to a hairpin structure stabilized by CG and GT base pairs. Polymerase stop assays with a DNA template containing *hras*-1show that Taq polymerase is arrested in the presence of phthalocyanines, indicating that the G-quadruplex formed by *hras*-1 is less stable than that formed by *hras*-2.
(TIF)

Figure S2 CD spectra of the *hras*-1 and *hras*-2 mutants. CD spectra in 50 mM Tris-HCl pH 7.4, 100 mM KCl of *hras*-1, *hras*-1 mut (5′-TC**GGG**TTGC**GGG**CGCA**GGG**CAC**CTG**CG), *hras*-2 and *hras*-2 mut (5′-CGAGGCCG**GTG**C**GGT**GCGG**GGG**C**GGG**GGCGCGCGGT). Cuvette 0.5 cm, DNA concentration 6 µM.
(TIF)

Figure S3 EMSA showing the binding between recombinant MAZ-GST and Sp1-GST with duplexes *hras*-1 and *hras*-2. (a) radiolabelled *hras*-1 duplex, 15 nM incubated for 30 min with 0, 0.5, 1, 1.5, 2 and 2.5 µg MAZ-GST; (b) radiolabelled *hras*-2 duplex, 15 nM incubated for 30 min with 0, 0.5, 1, 1.5, 2 and 2.5 µg MAZ-GST; (c) radiolabelled *hras*-2 duplex, 15 nM incubated for 30 min with 0, 0.5, 1 and 2.5 µg Sp1-GST.
(TIF)

Figure S4 SDS-PAGE of eluate from Glutathione Sepharose 4B column loaded with protein extract of BL21 bacteria transformed with a plasmid encoding for MAZ-GST or Sp1-GST (a) lane 1: bacterial extract; lane 2: proteins that did not bind the resin (flow through); lane 3, fraction eluted with 10 mM glutathione; (b) lane 1: molecular weights; lane 2: bacterial extract; lane 3: proteins that did not bind the resin (flow through); lane 4, column wash; lane 5: 1^st^ elution with 10 mM glutathione; lane 6: 2^nd^ elution with 10 mM glutathione.
(TIF)

Figure S5 Silencing in HeLa cells of MAZ and Sp1 by commercial shRNAs. HeLa cells have been treated with MAZ shRNA or Sp1 shRNA complexed with Metafectene. As control we treated HeLa cells with control shRNA. MAZ and Sp1 specific shRNA and control shRNA have been purchased from Santa Cruz Biotechnology (USA). After 48 h, total RNA was extracted, transformed in cDNA and used for real-time experiments. The levels of MAZ and Sp1 transcripts have been measured and reported in graph relatively to the expression of three housekeeping genes: *GAPDH*, β2- microglobulin, hypoxanthine ribosyl transferase.
(TIF)

Figure S6 (a) SDS-PAGE of fractions eluted from a Glutathione Sepharose 4B column loaded with protein extract obtained from non-transformed BL21 DE3 plysS bacteria (lanes 5 and 6). Fractions eluted with 10 mM reduced glutathione do not contain bacterial proteins bound non-specifically to the resin; (b) (top) FRET melting of 200 nM quadruplex *hras*-1 in 50 mM Tris-HCl, pH 7.4, 50 mM KCl, 50 µM Zn-acetate in the presence of FPLC purified GST at DNA∶protein ratios of 1∶0, 1∶1 and 1∶5; (bottom) FRET melting of 200 nM quadruplex *hras*-2 in 50 mM Tris-HCl, pH 7.4, 100 mM NaCl, 50 µM Zn-acetate in the presence of FPLC purified GST at DNA∶protein ratios of 1∶0, 1∶1 and 1∶5.
(TIF)

Figure S7 (Top) putative structures of the designed G4-decoys. The yellow rectangles represent the TINA unit (P); (bottom) Structure of the TINA unit covalently inserted in the decoy oligonucleotides.
(TIF)

Figure S8 Proliferation assay with T24 cells untreated and treated with 800 nM G4-decoys **3**,**4**,**5** (mimicking *qhras*-1) and G4-decoys **6**,**7**,**8** (mimicking *qhras*-2). Decoy **637** is a random sequence containing one P unit. Two doses, one 48 h after the other, of 800 nM G4-decoys mixed with polyethylenimine have been delivered to T24 cells. Viable cells, measured by a resazurin assay, have been performed at increasing times from 1^st^ treatment.
(TIF)
