# Supplementary figures and images for: G4-DNA Formation in the HRAS Promoter and Rational Design of Decoy Oligonucleotides for Cancer Therapy
Source: PLoS One. 2011 Sep 8;6(9):e24421. doi: 10.1371/journal.pone.0024421 (PMC3169596; doi:10.1371/journal.pone.0024421)

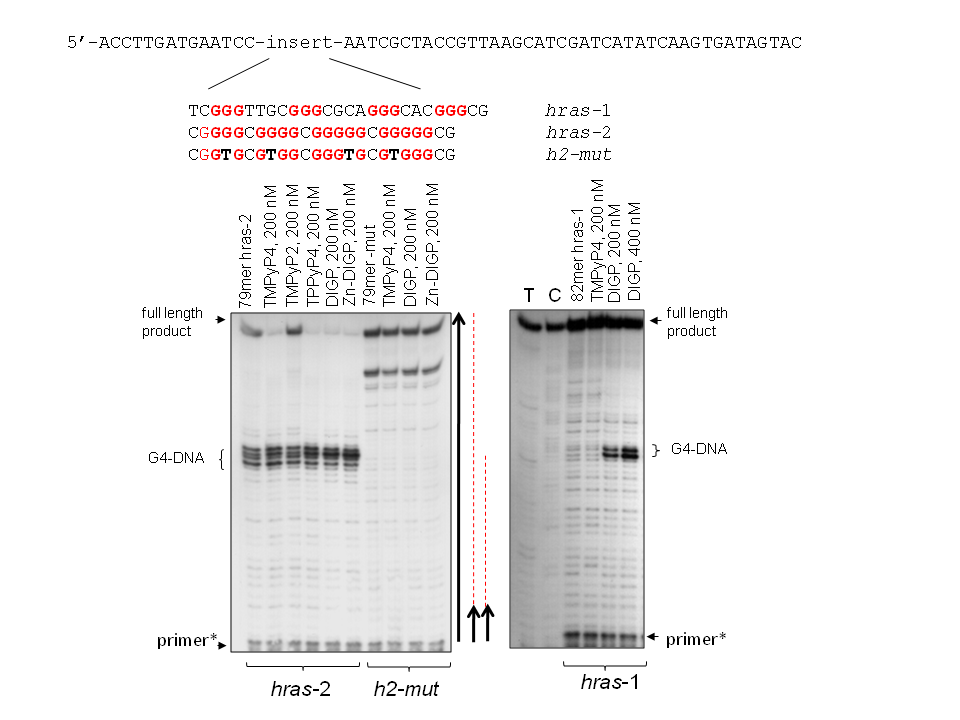

Supplement: Figure S1 — Polymerase stop assays with a wild-type DNA template containing hras-1 or hras-2 and a mutant template in which four G→T point mutations were introduced in sequence hras-2 to abrogate quadruplex formation. Taq polymerase is arrested at the 3′ end of hras-2, before the first run of guanines, in keeping with the formation of a quadruplex structure by hras-2 (experimental conditions: 37°C, 140 mM KCl, 50 mM Tris-HCl pH 7.4) When the DNA template is incubated with G4-DNA ligands that stabilize quadruplex DNA, Taq polymerase is completely arrested and only the truncated product is produced. This is observed with porphyrin TMPyP4 and guanidine phthalocyanines DIGP and Zn-DIGP at r = 4 (r = [ligand]/[template]). Instead, TMPyP2, which does not bind to G4-DNA does not affect the processivity of Taq polymerase. When the experiment is performed with the mutated template, Taq polymerase does not stop at the G-element and full product is observed. A longer truncated product is observed with the mutated template, probably due to a hairpin structure stabilized by CG and GT base pairs. Polymerase stop assays with a DNA template containing hras-1show that Taq polymerase is arrested in the presence of phthalocyanines, indicating that the G-quadruplex formed by hras-1 is less stable than that formed by hras-2. (TIF) [file pone.0024421.s001.tif]

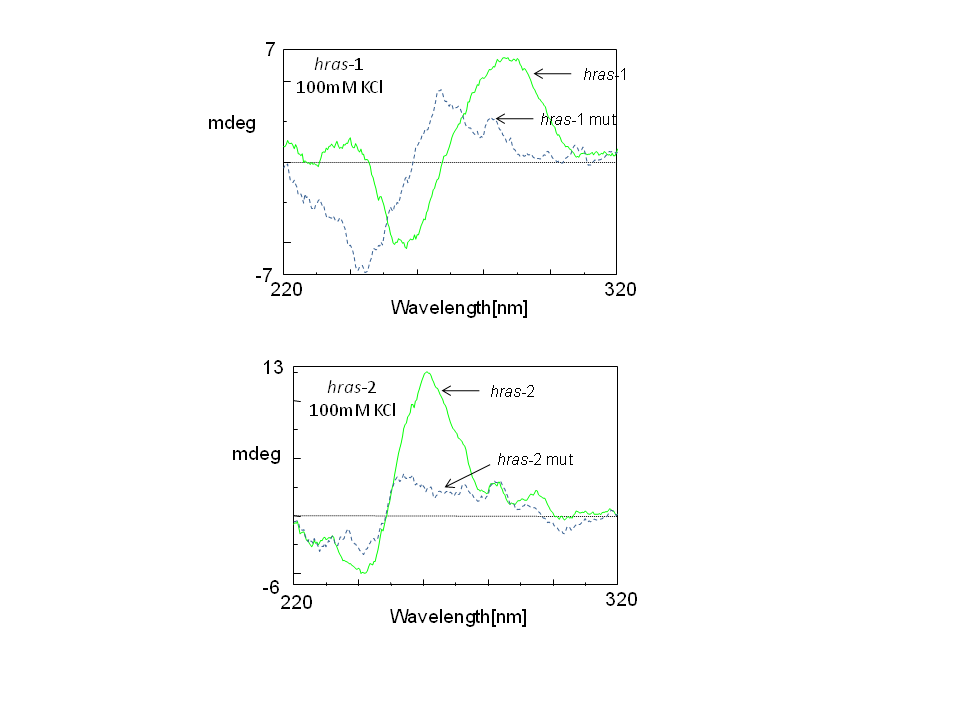

Supplement: Figure S2 — CD spectra of the hras-1 and hras-2 mutants. CD spectra in 50 mM Tris-HCl pH 7.4, 100 mM KCl of hras-1, hras-1 mut (5′-TCGGGTTGCGGGCGCAGGGCACCTGCG), hras-2 and hras-2 mut (5′-CGAGGCCGGTGCGGTGCGGGGGCGGGGGCGCGCGGT). Cuvette 0.5 cm, DNA concentration 6 µM. (TIF) [file pone.0024421.s002.tif]

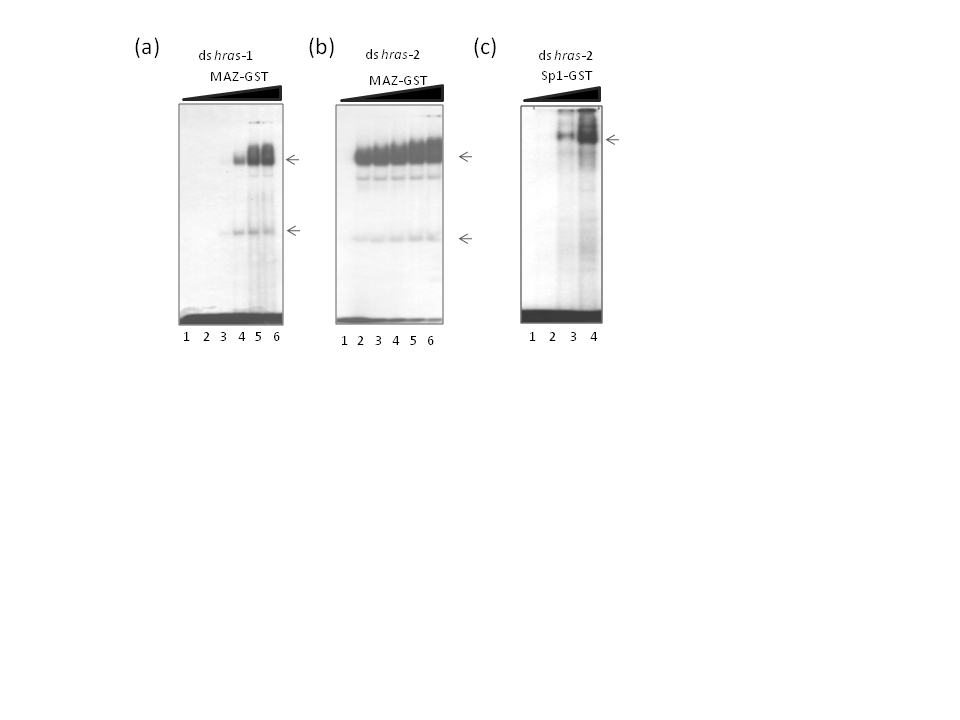

Supplement: Figure S3 — EMSA showing the binding between recombinant MAZ-GST and Sp1-GST with duplexes hras-1 and hras-2. (a) radiolabelled hras-1 duplex, 15 nM incubated for 30 min with 0, 0.5, 1, 1.5, 2 and 2.5 µg MAZ-GST; (b) radiolabelled hras-2 duplex, 15 nM incubated for 30 min with 0, 0.5, 1, 1.5, 2 and 2.5 µg MAZ-GST; (c) radiolabelled hras-2 duplex, 15 nM incubated for 30 min with 0, 0.5, 1 and 2.5 µg Sp1-GST. (TIF) [file pone.0024421.s003.tif]

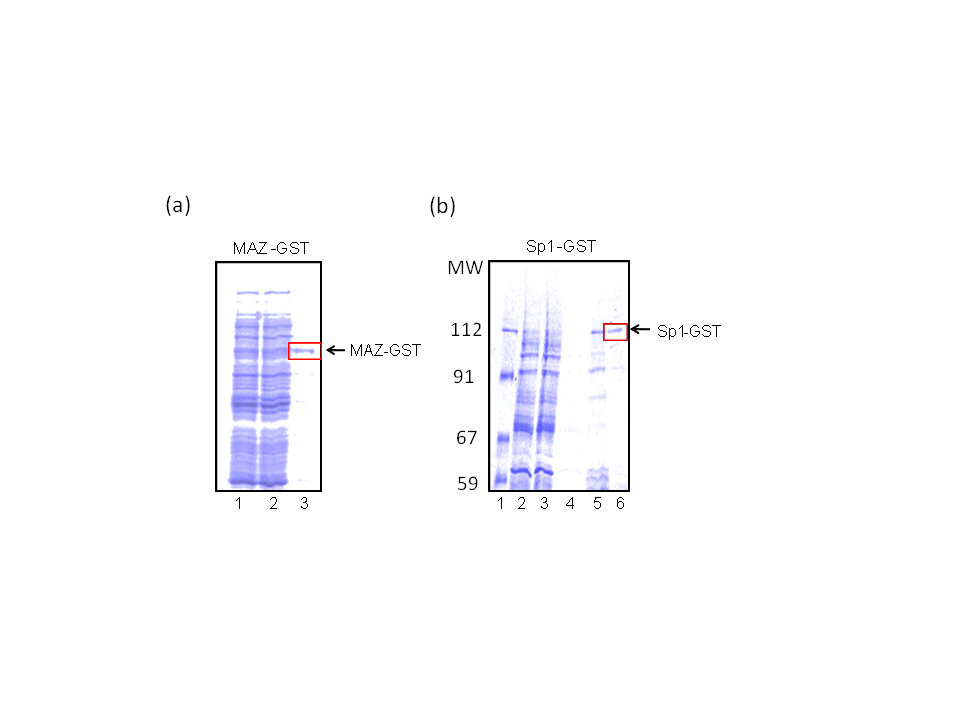

Supplement: Figure S4 — SDS-PAGE of eluate from Glutathione Sepharose 4B column loaded with protein extract of BL21 bacteria transformed with a plasmid encoding for MAZ-GST or Sp1-GST (a) lane 1: bacterial extract; lane 2: proteins that did not bind the resin (flow through); lane 3, fraction eluted with 10 mM glutathione; (b) lane 1: molecular weights; lane 2: bacterial extract; lane 3: proteins that did not bind the resin (flow through); lane 4, column wash; lane 5: 1st elution with 10 mM glutathione; lane 6: 2nd elution with 10 mM glutathione. (TIF) [file pone.0024421.s004.tif]

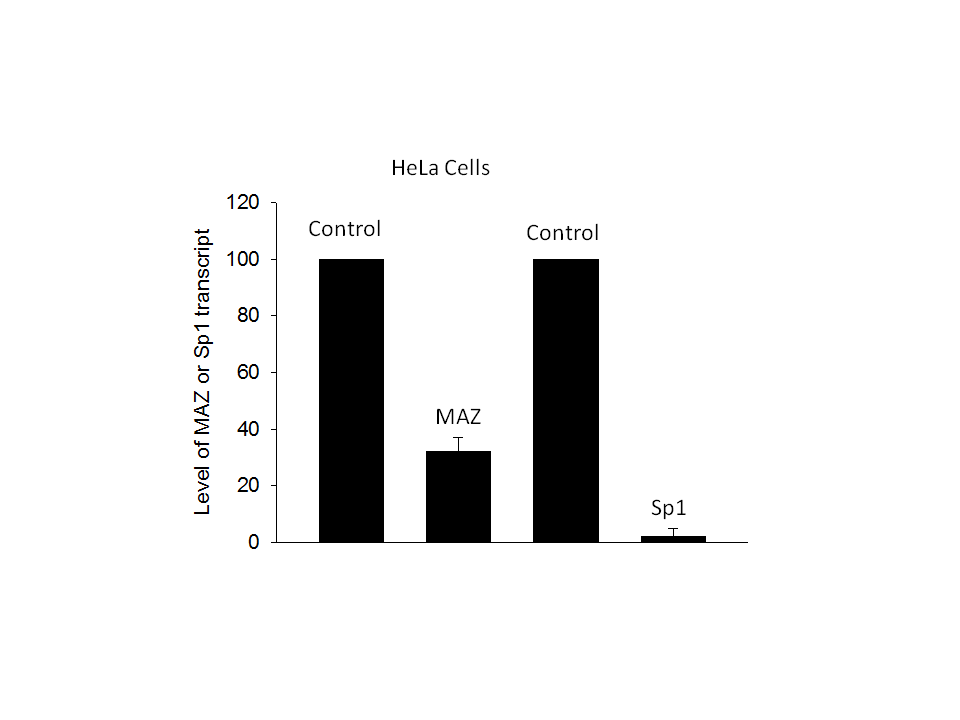

Supplement: Figure S5 — Silencing in HeLa cells of MAZ and Sp1 by commercial shRNAs. HeLa cells have been treated with MAZ shRNA or Sp1 shRNA complexed with Metafectene. As control we treated HeLa cells with control shRNA. MAZ and Sp1 specific shRNA and control shRNA have been purchased from Santa Cruz Biotechnology (USA). After 48 h, total RNA was extracted, transformed in cDNA and used for real-time experiments. The levels of MAZ and Sp1 transcripts have been measured and reported in graph relatively to the expression of three housekeeping genes: GAPDH, β2- microglobulin, hypoxanthine ribosyl transferase. (TIF) [file pone.0024421.s005.tif]

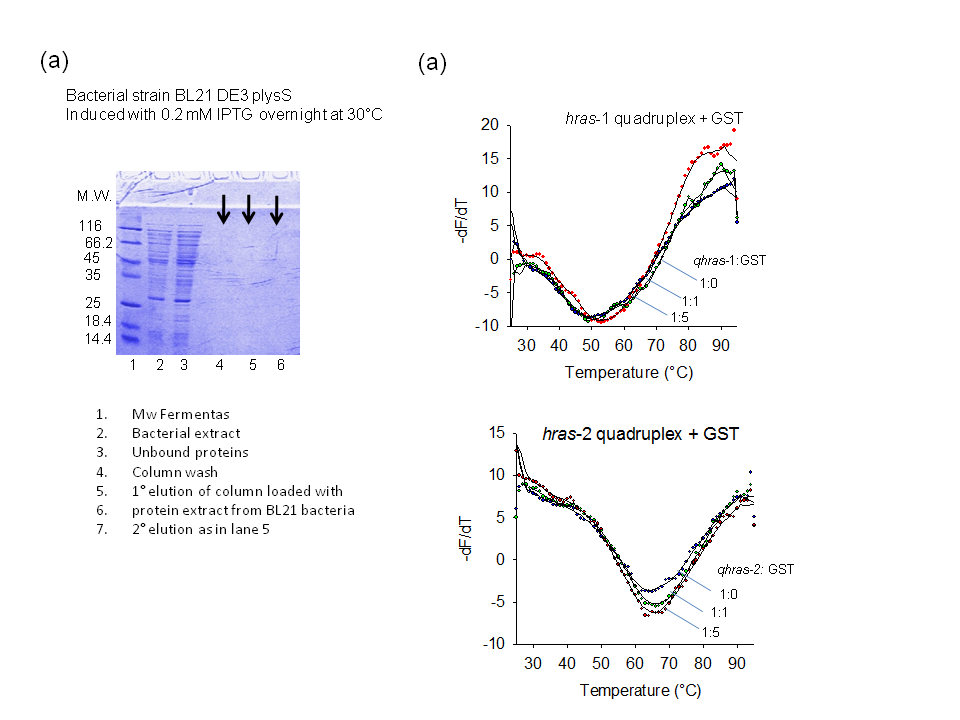

Supplement: Figure S6 — (a) SDS-PAGE of fractions eluted from a Glutathione Sepharose 4B column loaded with protein extract obtained from non-transformed BL21 DE3 plysS bacteria (lanes 5 and 6). Fractions eluted with 10 mM reduced glutathione do not contain bacterial proteins bound non-specifically to the resin; (b) (top) FRET melting of 200 nM quadruplex hras-1 in 50 mM Tris-HCl, pH 7.4, 50 mM KCl, 50 µM Zn-acetate in the presence of FPLC purified GST at DNA∶protein ratios of 1∶0, 1∶1 and 1∶5; (bottom) FRET melting of 200 nM quadruplex hras-2 in 50 mM Tris-HCl, pH 7.4, 100 mM NaCl, 50 µM Zn-acetate in the presence of FPLC purified GST at DNA∶protein ratios of 1∶0, 1∶1 and 1∶5. (TIF) [file pone.0024421.s006.tif]

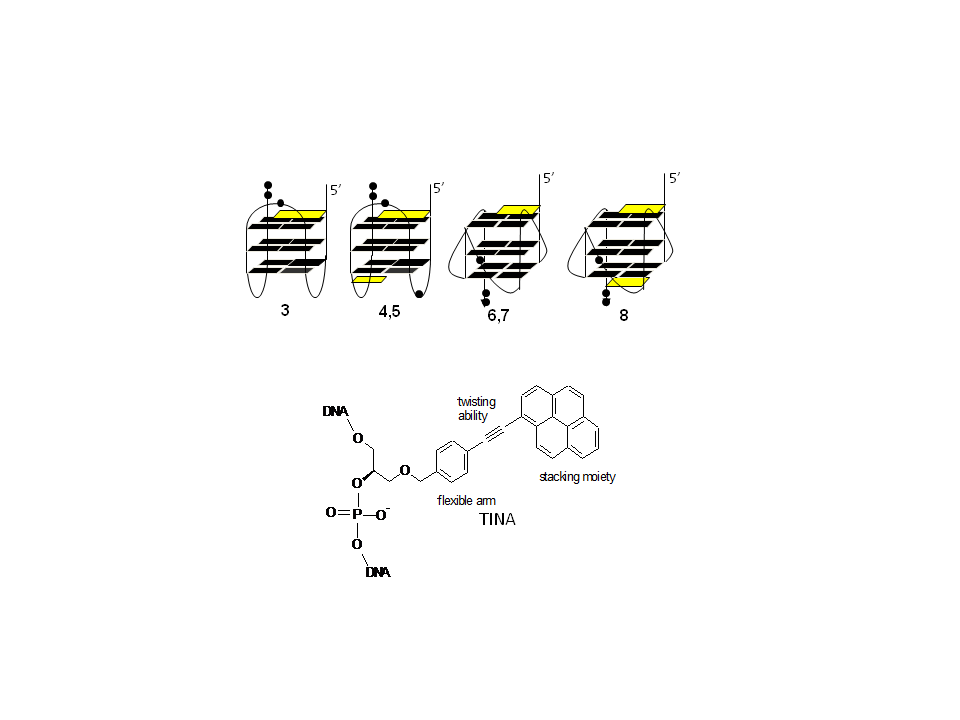

Supplement: Figure S7 — (Top) putative structures of the designed G4-decoys. The yellow rectangles represent the TINA unit (P); (bottom) Structure of the TINA unit covalently inserted in the decoy oligonucleotides. (TIF) [file pone.0024421.s007.tif]

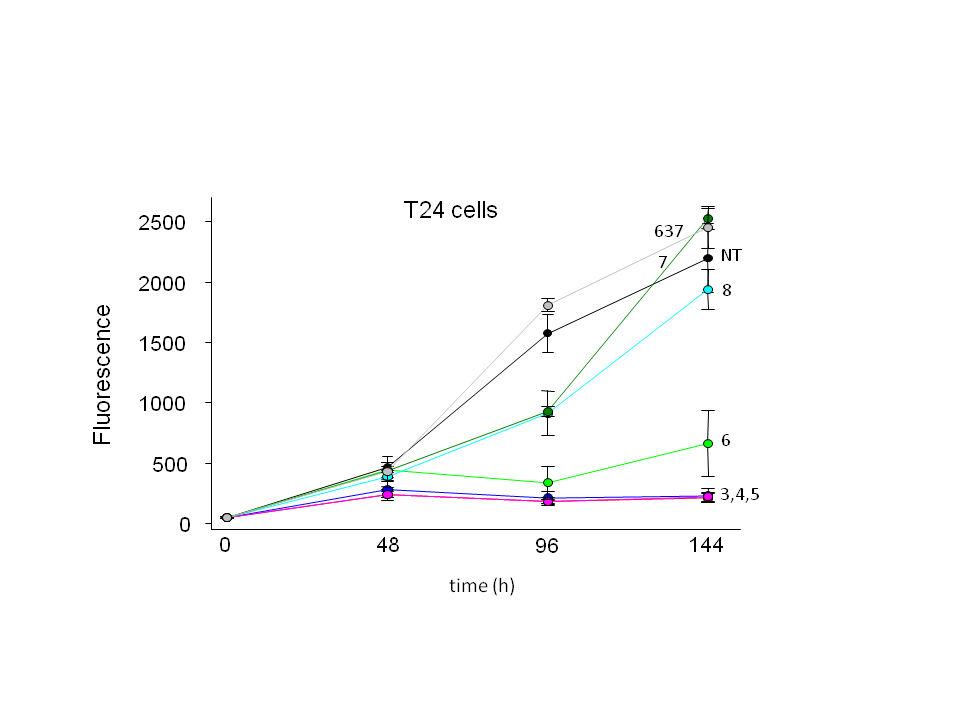

Supplement: Figure S8 — Proliferation assay with T24 cells untreated and treated with 800 nM G4-decoys 3,4,5 (mimicking qhras-1) and G4-decoys 6,7,8 (mimicking qhras-2). Decoy 637 is a random sequence containing one P unit. Two doses, one 48 h after the other, of 800 nM G4-decoys mixed with polyethylenimine have been delivered to T24 cells. Viable cells, measured by a resazurin assay, have been performed at increasing times from 1st treatment. (TIF) [file pone.0024421.s008.tif]
